# Synthesis of new triazole derivatives and their potential applications for removal of heavy metals from aqueous solution and antibacterial activities

**DOI:** 10.3389/fchem.2024.1473097

**Published:** 2024-10-23

**Authors:** Chunyun Xu, Na Yang, Haichun Yu, Xiaojing Wang

**Affiliations:** Department of Dermatology, Maternity and Child Health Hospital of Qinhuangdao, Qinhuangdao, China

**Keywords:** 1,2,4-triazole derivatives, antibacterial activity, anti biofilm, anti inflammatory, metal ion detection

## Abstract

In this paper, triazole derivatives were prepared by a three-step mild reaction using carbon disulfide as starting material. In face of microbial threats, we found that compound 3-cyclopropyl-[1,2,4]triazolo [3,4-b][1,3,4]thiadiazole-6-thiol (**C2**) has good antibacterial activity, inhibition and clearance ability against biofilms, low hemolytic activity and toxicity, good anti-inflammatory activity. At the same time, we found that **B** and **C** series compounds have good metal ion scavenging ability, with removal rates of **C** series ranging from 47% to 67% and **B** series ranging from 67% to 87%.

## Graphical Abstract

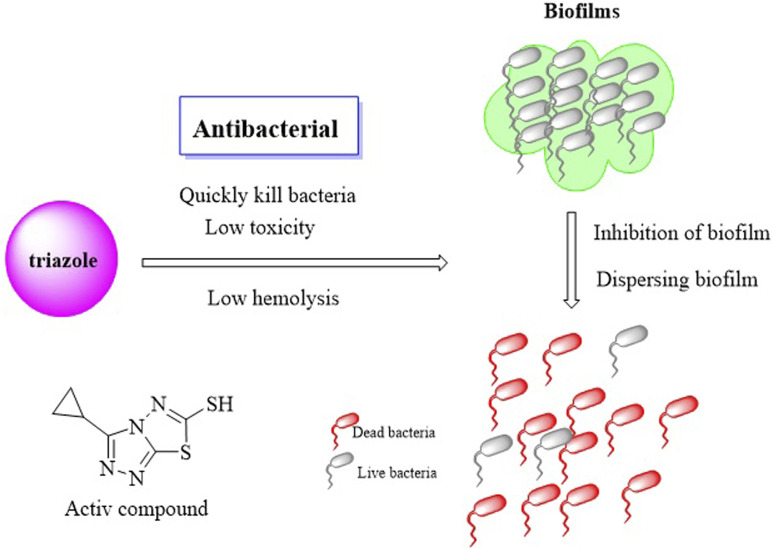

## 1 Introduction

Bacterial or fungal infections pose a huge threat to human health, animal husbandry, and other industries. While antibiotics exert their antibacterial effects, pathogenic bacteria are also constantly adapting to antibiotics and developing resistance (Buder et al., 2019; Jean et al., 2022). Traditional drugs such as beta lactams, aminoglycosides, tetracyclines, macrolides, glycopeptides, quinolones, and so on inhibit bacterial growth by interfering with the components of intracellular biochemical pathways. But smart bacteria develop resistance through several pathways, including bypassing inhibitory steps, changing the site of action, efflux mechanisms, target mutations, and changes in cell wall permeability (Scott et al., 2008). Specifically, Aminoglycoside-modifying enzymes, for example, acetyltransferases, phosphotransferases and nucleotidyltransferases; Production of β-lactamases; reduced permeability and increased efflux; Modification or removal of lipid A; intermediate susceptibility phenotype conferred by mutations leading to thickened membrane and low permeability; rRNA methyltransferases, which methylate 23S rRNA; Mutations in DNA gyrase or topoisomerase IV; Mutations in the drug target rpoB; protein-mediated ribosome protection, etc., (Burki, 2018). In addition, the abuse of drugs has led to the production of some super bacteria. In order to overcome bacterial resistance, there is an urgent need for new antimicrobial drugs (Burki, 2018; Thanh Dong et al., 2020).

**FIGURE 1:**
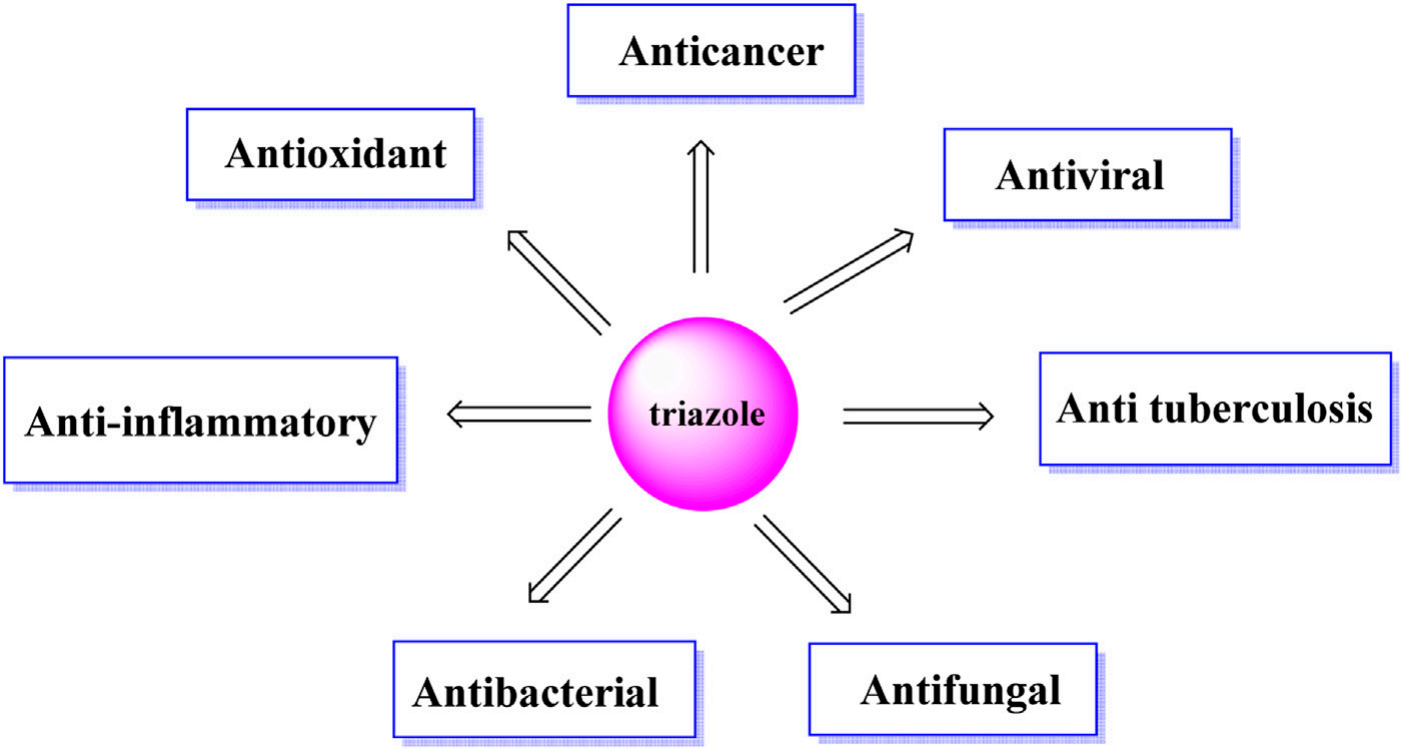
Significant biological activities of triazole.

Triazole compounds have become a very active research field due to their extensive potential applications in pharmaceuticals, pesticides, materials, artificial receptors, supramolecular recognition, and biomimetic simulations (Sharma et al., 2023). The three nitrogen atoms in the triazole ring and their unique five membered aromatic nitrogen heterocyclic structure make the triazole ring susceptible to various non covalent interactions, such as hydrogen bonding, coordination with metal ions, hydrophobic interactions, π - π stacking, electrostatic interactions, etc., Therefore, triazole ring can easily bind to various enzymes and receptors in organisms, exhibiting various biological activities (Kaproń et al., 2019; Kaur and Chawla, 2017). Activities such as anticancer (El-Sherief et al., 2018; Shahzad et al., 2019), antiviral (Feng et al., 2020; Seliem et al., 2021), anti tuberculosis (Xu et al., 2017b; Zhang S. et al., 2017), antifungal (Jin et al., 2018; Xu et al., 2011), antibacterial and so on (Figure 1) (Eswaran et al., 2009; Fan et al., 2018). More importantly, the triazole ring is also commonly used as a isostere and is widely used to replace functional groups such as imidazole, benzimidazole, oxazole, pyrazole, thiazole, amide, etc., In the design and development of new drugs, playing an important role in improving the biological activity of compounds. Therefore, triazole rings are widely used to construct various functional molecules, especially in the development of their pharmaceutical applications, which is currently one of the key areas of drug research and development (Hu et al., 2017; Xu et al., 2017a). Therefore, the combination of 1,2,3-triazole with various molecules may provide efficient antibacterial candidates. So we synthesized a series of triazole derivatives and tested their antimicrobial activity against four species of Aureus and *Escherichia coli*. Also toxicity and hemolysis of the drugs are important indicators for evaluation of the drug and we also tested them. As the formation of biofilm by bacteria increases the bacterial resistance, we also conducted biofilm consistency and clearance experiments to determine the resistance and bactericidal profiles of the active compounds. When infection occurs, it is often accompanied by inflammation, so we tested the anti-inflammatory activity of the active compounds. Finally, for subsequent in-depth study of the antimicrobial mechanism, we docked with antimicrobial potential target proteins by molecular docking.

Although some pollutants have been registered as newly emerging pollutants in water, there have been no reports on the application of triazole compounds in water treatment processes. According to the World Health Organization (WHO) and the United States Environmental Protection Agency (EPA), when metals and heavy metal ions in water exceed the maximum pollution level, it can lead to environmental problems (Bochynska et al., 2024; Ding et al., 2021; Li et al., 2018; Yan et al., 2024). Due to these facts, this article measured the efficiency of synthesized triazole derivatives in removing metals and heavy metal ions from water.

In conclusion, in this paper, the antimicrobial agent triazole heterocycle was synthesized from carbon disulfide and all compounds were detected by ^1^H NMR (Nuclear Magnetic Resonance) and ^13^C NMR. The synthesized triazole derivatives have a high removal rate Pb^2+^, Cd^2+^, Ca^2+^ and Mg^2+^, good antibacterial effect, good anti-inflammatory, and good biofilm inhibition and biofilm dispersal activity.

## 2 Results and discussion

### 2.1 Chemical synthesis

**SCHEME 1:**
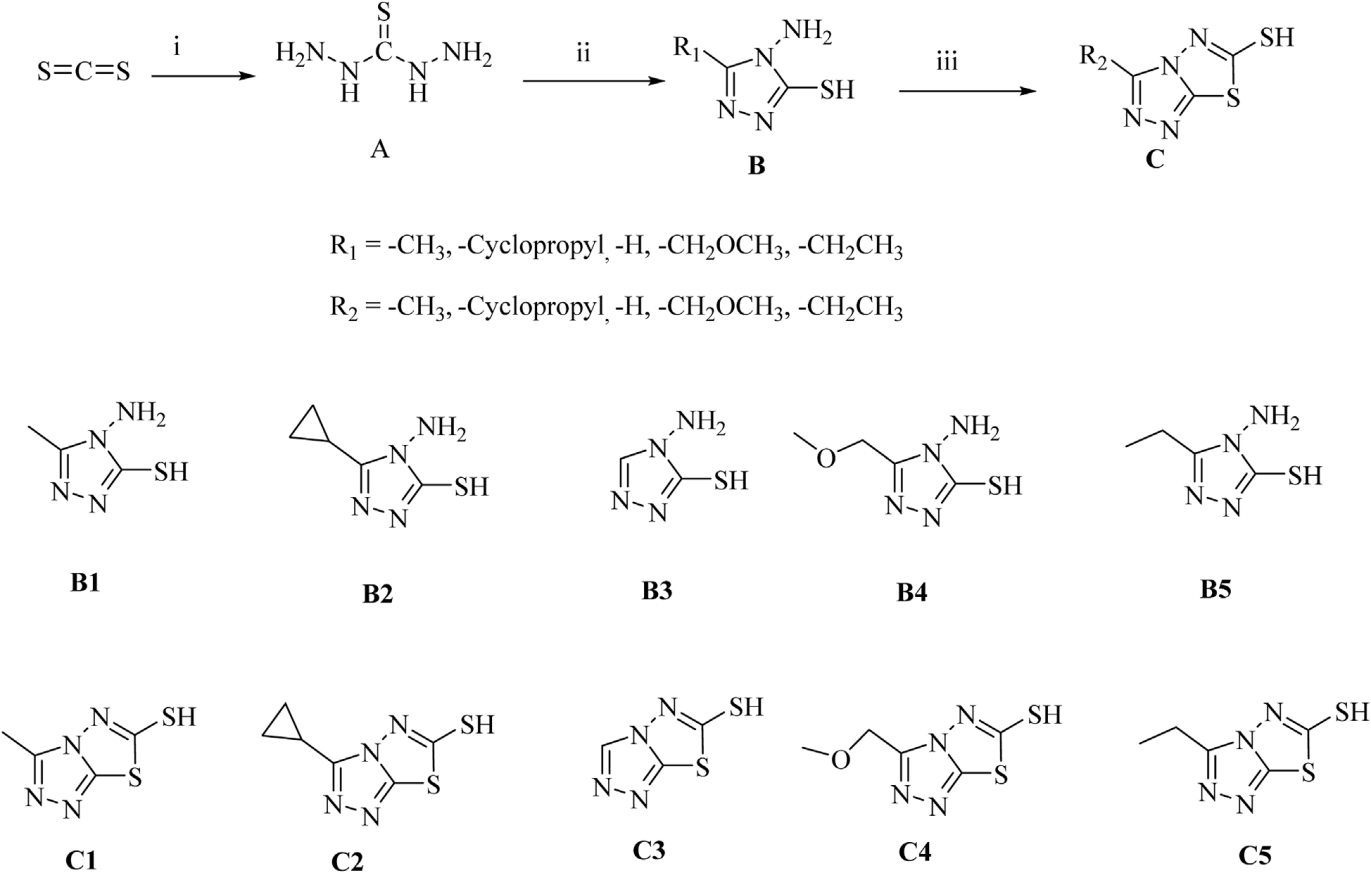
Synthesis of Triazole Derivatives. Conditions and reagents: (i) NH_2_NH_2*_H_2_O and CS_2_, H_2_O, reflux, 10 h, yield 53%; (ii) Different substituted carboxylic acids, reflux, yield 56%–78%; (iii) CS_2_, KOH, CH_3_OH, reflux, yield 46%–65%.

We prepared our products (**B** and **C**) by a three-step and mild reaction conditions using carbon disulfide as starting material (Scheme 1). All compounds were characterized by NMR and tested for melting points. It is worth noting that all of our products can be obtained by recrystallisation, with yields above 46% in each step, avoiding the tedious steps of silica gel column purification, and having certain prospects for industrial application.

### 2.2 The antibacterial activity of the compounds

#### 2.2.1 Determination of minimum inhibitory concentration

Minimum Inhibitory Concentration Determination study on Triazole compounds derivatives showed that these compounds have antimicrobial activity (Tian et al., 2023). In this study, the Triazole compounds were evaluated *in vitro* antibacterial activity Minimum inhibitory concentration (MIC) reference strains of Gram-positive bacteria—*S. aureus ATCC 29312, S. aureus ATCC 43300, S. aureus ATCC 33731, S. aureus MRSA*, and Gram-negative bacterial strain of *E*. *coli ATCC 25922* using broth microdilution method with drug concentrations from 256 μg/mL to 0.5 μg/mL (Baquer et al., 2021). The results of the antimicrobial activity of the tested compounds are presented in Table 1. Among the tested compounds, **C2** showed good bacteriostatic activity against *E. coli* with a MIC of 2 μg/mL. In addition, compound **C2** exhibited inhibitory activity against all tested Gram positive strains at MIC of 32 μg/mL.

**TABLE 1:** Minimum inhibitory concentration (MIC) [µg/mL] of triazole derivatives on reference bacterial strains.

Compound	MIC (µg/mL)^a^				
	S. aureus ATCC 29213	S. aureus ATCC 43300	S. aureus ATCC 33731	S. aureus MRSA	E. coli ATCC 25922
Vancomycin^b^	2	2	2	2	>256
C1	>256	>256	>256	>256	>256
C2	≥32	≥32	≥32	≥32	≥2
C3	≥128	≥128	≥128	≥64	≥32
C4	>256	>256	>256	>256	>256
C5	>256	>256	>256	>256	>256

^a^ The minimum inhibitory concentration (MIC) is the lowest concentration that completely inhibits microbial growth after 16–24 h. Each experiment was repeated three times.

^b^ vancomycin is a clinical drug against Gram-positive bacteria.

#### 2.2.2 Time-killing curve determinations

**FIGURE 2:**
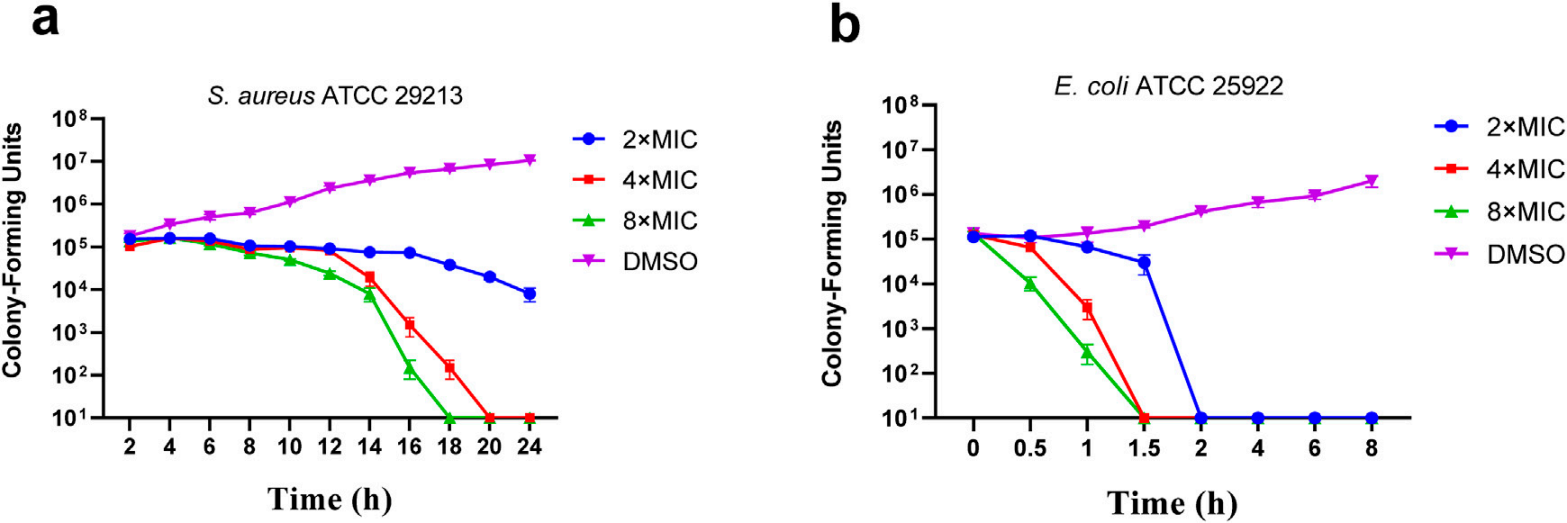
Time-kill kinetics of **C2** against *S. aureus* ATCC 29213 **(A)** and *E. coli* ATCC 25922 **(B)**. Data are presented as means ± SEM (Standard Error of Mean) from three independent experiments.

Time killing curves were determined by counting bacterial colonies at different time points to determine whether the compounds were bactericidal or not. The time–kill curve assay of compound **C2** for *E. coli ATCC 25922* and *S. aureus ATCC 29213* were carried out subsequently with DMSO as a negative control. The results of the time-kill curves of **C2** against *S. aureus ATCC 29213* was shown in Figures 2A, E *Coli ATCC 25922* was shown in Figure 2B. The results show that the growth of *E. coli ATCC 25922* and *S. aureus ATCC 29213* can be completely inhibited at 2 × MIC and at 8 × MIC respectively.

#### 2.2.3 Drug resistance study

**FIGURE 3:**
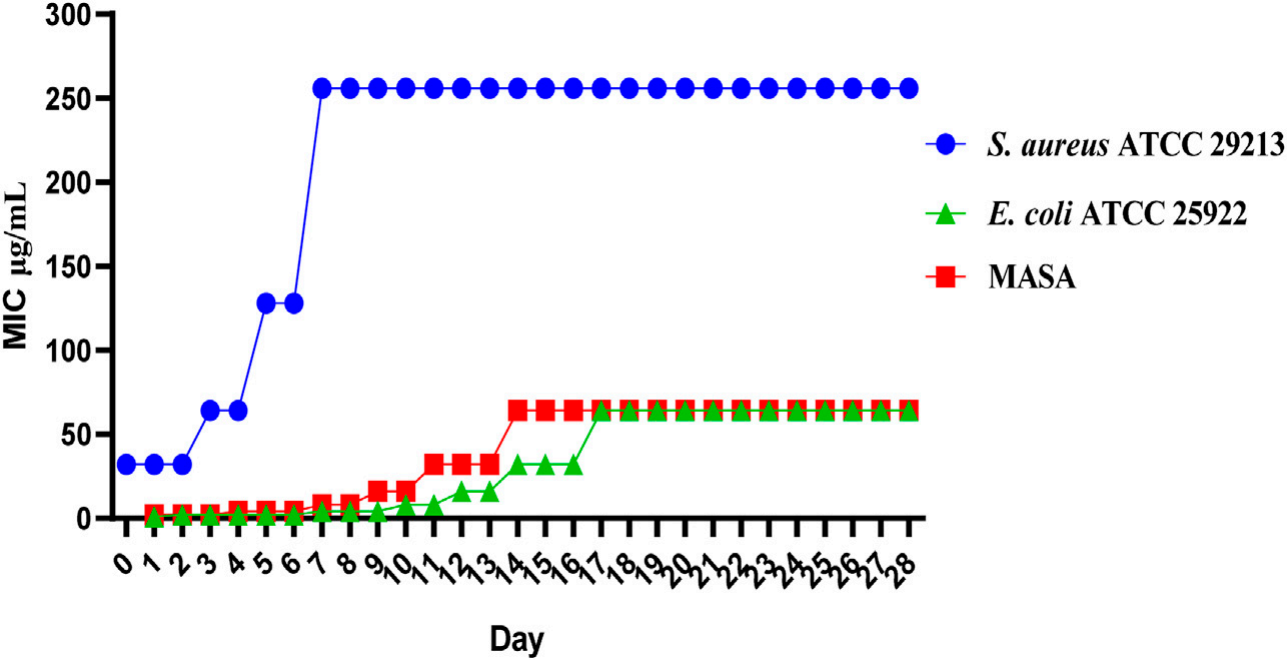
Resistance development of **C2**. Data are presented as means ± SEM from three independent experiments.

The results of compounds **C2** on drug resistance study suggested that **C2** has a low spontaneous frequency of resistance on *E. coli ATCC 25922, S. aureus ATCC 29213* and MASA. As shown in Figure 3, *E. coli ATCC 25922, S. aureus ATCC 29213* and MASA did not produce resistant mutants at sublethal concentrations of 0.5 × MIC over a period of 28 days, and the MIC values did not increase more than 8-fold after 28 passages. These results indicate that **C2** can kill bacteria effectively and avoid the development of drug resistance.

### 2.3 The toxicity of the compounds

#### 2.3.1 Hemolysis assay

**FIGURE 4:**
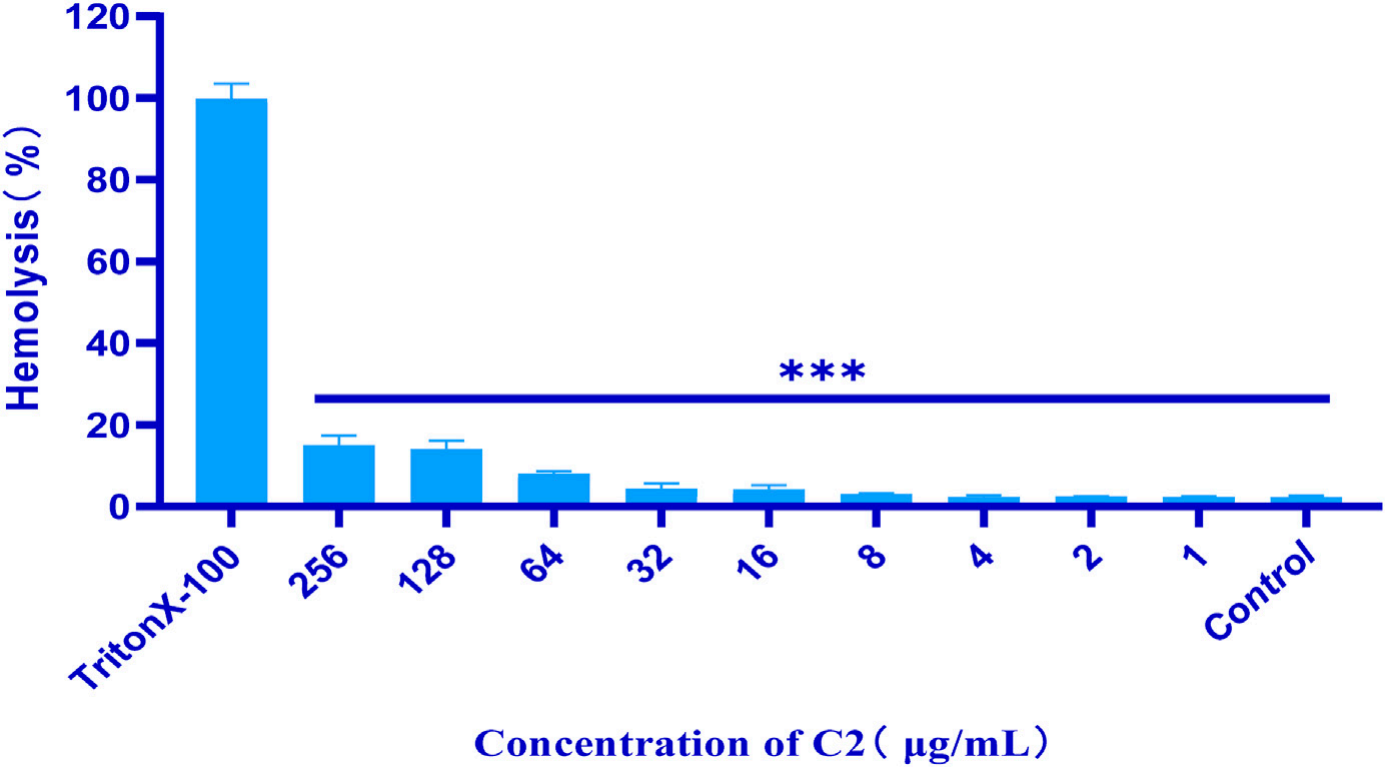
Percentage of hemolysis of rabbit blood cells at various **C2** concentrations. Difference is considered significant at ^*^p < 0.05, ^**^p < 0.01, ^***^p < 0.001. Data are presented as means ± SEM from three independent experiments.

Most researchers study the hemolytic (cytotoxic) effects of compounds on isolated red blood cells (Farag and Alagawany, 2018). Such research does not allow for a thorough evaluation of the activity of compounds and the impact of other blood components on red blood cell lysis. In order to conduct antibacterial stability testing in our work, we first conducted hemolysis tests on all tested compounds. To establish a positive control, 1% Triton X-100 was used, whereas sterile PBS served as the negative control. The results of this measurement are shown in Figure 4. After incubation at 37°C for 24 h, the hemolysis assay of all test compounds were observed in 4% rabbit red blood cells *in vitro*. The test compound with a concentration of 1–256 μ g/mL did not show hemolytic characteristics. This indicates that compound **C2** did not cause hemolysis of rabbit erythrocytes, even at its active antibacterial concentration.

#### 2.3.2 Cell cytotoxicity assay

**FIGURE 5:**
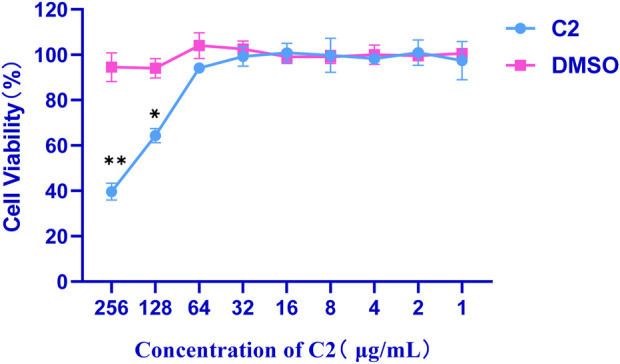
Cytotoxicity of compound **C2** against Vero cells after 24 h. Difference is considered significant at ^*^p < 0.05, ^**^p < 0.01, ^***^p < 0.001. Data are presented as means ± SEM from three independent experiments.

Cytotoxicity of the active compound **C2** was evaluated against african green monkey kidney cells line (VERO cell) using the CCK8 assay (Wang et al., 2023). Briefly, selectivity toward prokaryotic cells was evaluated using VERO cell, to determine the potential toxic effect *in vitro*. As summarized in Figure 5, the maximal inhibitory concentration of these compounds against VERO cells was > 64 μg/mL, indicating that these compounds are not cytotoxic to VERO cells at concentrations as high as 64 μg/mL.

### 2.4 Inhibitory effects towards *Staphylococcus aureus* biofilm formation

**FIGURE 6:**
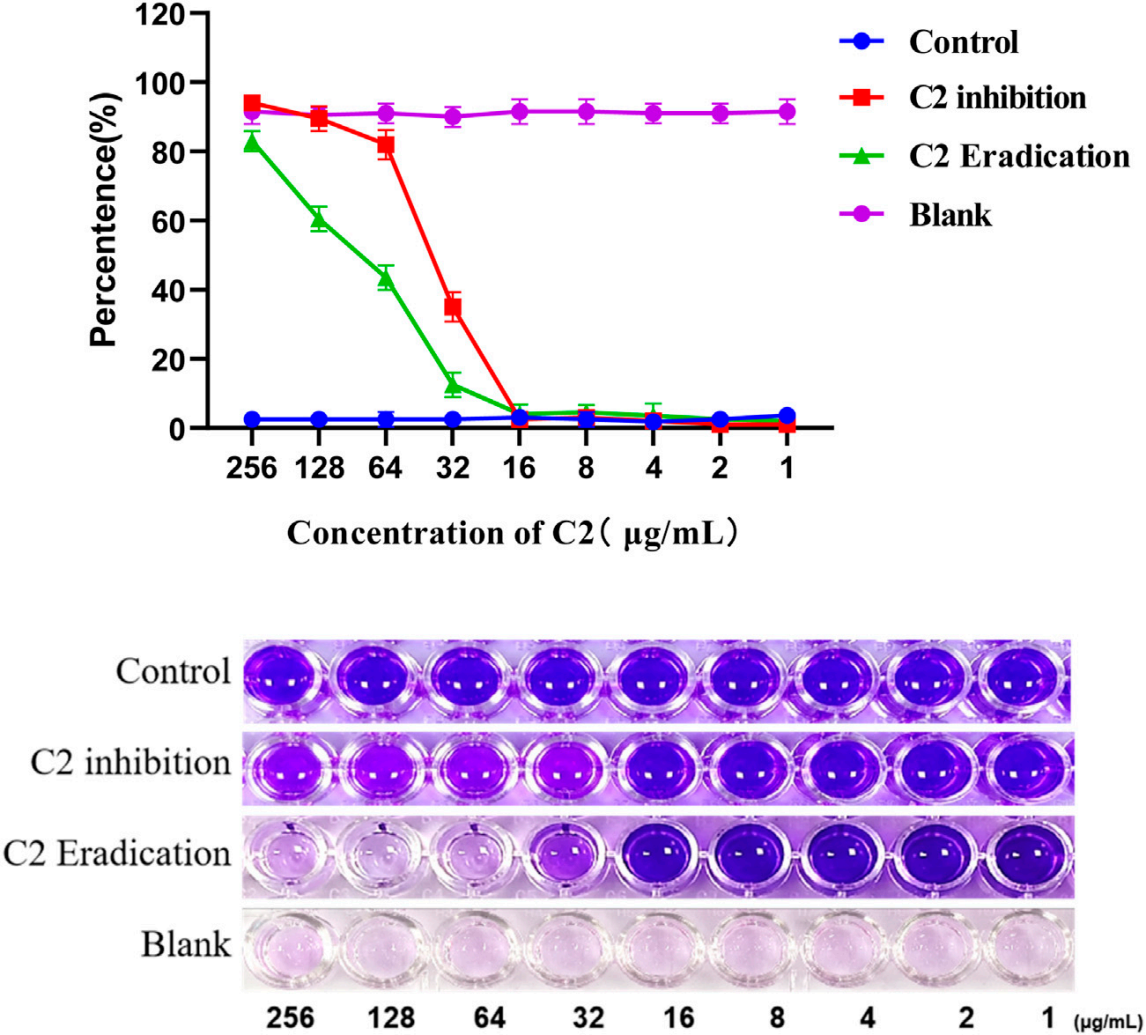
A dose-dependent response was observed in the biofilm inhibition and eradication activity of compound **C2** against *S. aureus* ATCC 29213. Difference is considered significant at ^*^ *p* < 0.05, ^**^p < 0.01, ^***^p < 0.001. Data are presented as means ± SEM from three independent experiments.

It is well known that microorganisms that can produce biofilms are one of the main factors leading to antibiotic resistance (Venkatesan et al., 2015). Therefore, many experiments have been carried out to overcome these serious problems by looking for new drugs that can prevent biofilm formation (Đukanović et al., 2022). *S. aureus* is one of the most common causes of biofilm related clinical infections. Next, we investigated whether compound **C2** inhibited the formation of *S. aureus* ATCC 29213 biofilm (Bauer et al., 2013). The crystal violet method was used to quantitatively analyze the biofilm, the inhibition percentage of compound 1–256 µ g/mL on *S. aureus ATCC 29213* biofilm is shown in the Figure 6. Compound **C2** showed significant biofilm inhibitory activity against *S*. aureus ATCC 29213 and compound **C2** showed 65.0% inhibition of biofilm at a concentration of 32 μg/mL.

Next, we studied whether **C2** compound could eradicate the biofilm formed by *S. aureus ATCC 29213*. The effect of biofilm eradication is expressed by the biofilm eradication concentration value, which is defined as the concentration of the compound required to eradicate the previous produced biofilm. After 24-hour treatment, compound **C2** showed a significant percentage of biofilm eradication activity against *S. aureus ATCC 29213*, as shown in Figure 6. For example, the eradication rate of compound 32 µ g/mL on biofilm is 87.5%. Compound **C2** showed 87.5% eradication on biofilm at a concentration of 32 μg/mL.

### 2.5 The anti-inflammatory activity of the compounds

**FIGURE 7:**
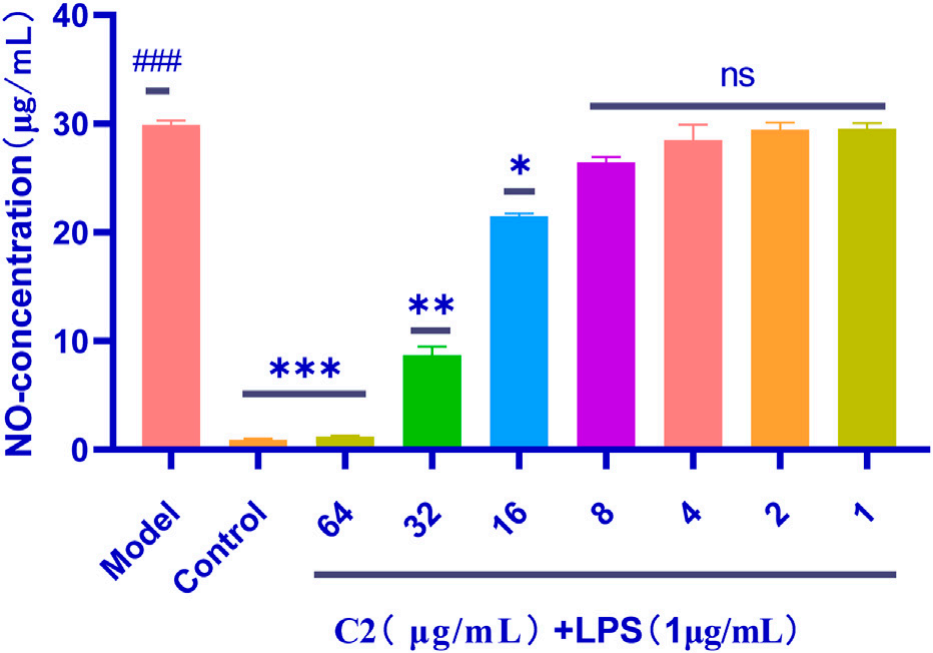
Anti-inflammatory activity of the **C2** compounds in RAW 264.7 macrophage cells was evaluated in the LPS-enhanced leukocyte migration assay. Compared with the blank control group, P< 0.001; compared with the LPS model group, ^*^p < 0.05, ^**^p < 0.01, ^***^p < 0.001, ns P> 0.5; ^###^p < 0.001 vs. control group. Data are presented as means ± SEM from three independent experiments.

Triazole compounds have anti-inflammatory effects in lipopolysaccharide stimulated mouse macrophages (RAW264.7) because they inhibit the protein expression of nitric oxide synthase (NOS) and cyclooxygenase-2 (COX-2) (Zhang H. J. et al., 2017), and the effect of active compound **C2** on nitric oxide levels. Therefore, compared to the NO produced by the control group, the use of LPS alone can significantly induce NO production. However, pre-treatment with the studied compounds would affect NO levels, which were significantly produced in LPS stimulated RAW 264.7 cells, as shown in the Figure 7. In addition, compared with LPS, compound **C2** has a significant inhibitory effect on NO production at concentrations as low as 16 μg/mL.

### 2.6 Screening of heavy metals and metal ion removal from aqueous solution

**FIGURE 8:**
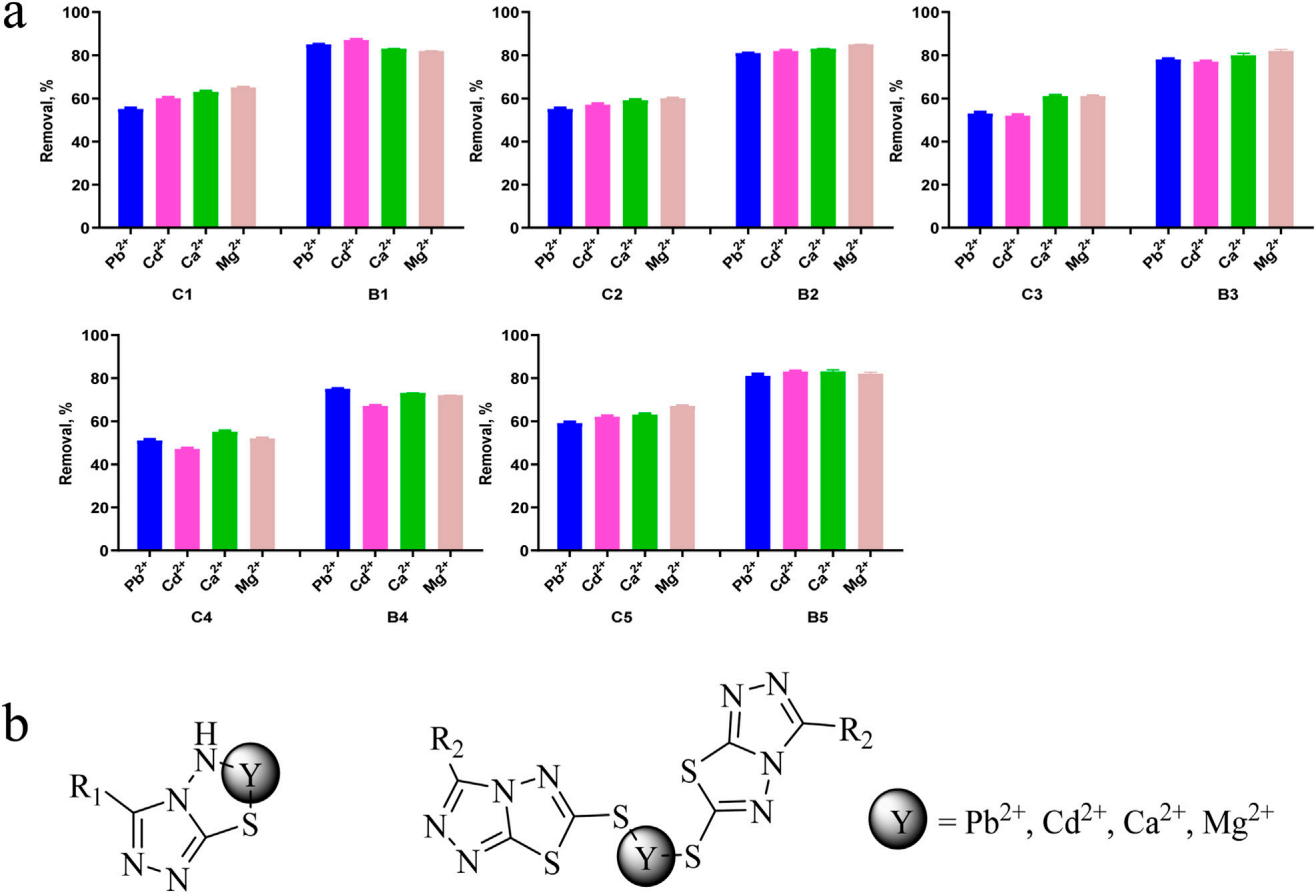
**(A)** Possible mechanism of metal and heavy metal ion removal, **(B)** Removal efficiencies of Pb^2+^, Cd^2+^, Ca^2+^ and Mg^2+^ using compounds. Data are presented as means ± SEM from three independent experiments.

In this work, the removal ability of compounds C1-C5 and B1-B5 on Pb^2+^, Cd^2+^, Ca^2+^ and Mg^2+^ in aqueous solutions was studied. As shown in Figure 8A, B-series compounds generally have stronger ability to remove metal ions than **C**-series compounds. The removal efficiency of C1-C5 for Pb^2+^, Cd^2+^, Ca^2+^ and Mg^2+^ in aqueous solution is 47%–67%, while the removal efficiency of B1-B5 for Pb^2+^, Cd^2+^, Ca^2+^ and Mg^2+^ in aqueous solution is 67%–87%. We speculate that the removal rate is related to the coordination form of the compound with metal ions. One possible mechanism (Figure 8B) is that in B-series compounds, an amino group forms a complex with a thiol and a metal ion, while C-series compounds require two compounds to bind with the metal ion, which reduces the clearance rate of C-series compounds towards the metal ion. Therefore, the efficiency of removing heavy metals and metal ions from aqueous solutions is influenced by the electronic effect of substituents.

The pH value is a key parameter for adsorbing heavy metals and metal ions. (Lingamdinne et al., 2017). Therefore, in this study, adsorption experiments were conducted at pH 6.0. Under acidic conditions with the pH value below 6, the studied compound is protonated, thereby reducing the interaction between heavy metals and metal ions with adsorbent molecules. In addition, when the pH value is greater than 6.0, metals and heavy metals can precipitate in the form of hydroxides [M(OH)_2_]. Therefore, when pH is chosen as 6.0, surface groups such as amino groups are deprotonated, which can chelate heavy metals and metal ions, as shown in Figure 8B. This ensures that only ions (Pb^2+^, Cd^2+^, Ca^2+^ and Mg^2+^) exist in the solution, eliminating the interference of hydroxide precipitation when the pH value is greater than 6.0. In addition, strong electrostatic repulsion occurring at lower pH values can achieve the highest removal efficiency.

Further research is needed on the mechanism of removing metals and heavy metal ions from water. For example, X-ray structural analysis of the formed complex is required, and FTIR spectroscopy is needed to determine the binding site by analyzing the functional groups attached to the metal/heavy metal ions in the synthesized compound. In addition, thermogravimetric analysis/differential thermal analysis (TGA/DTA) can be used to predict the loss of water molecules, as well as UV/Vis techniques to determine the stoichiometry of formed complexes.

### 2.7 Molecular docking

**FIGURE 9:**
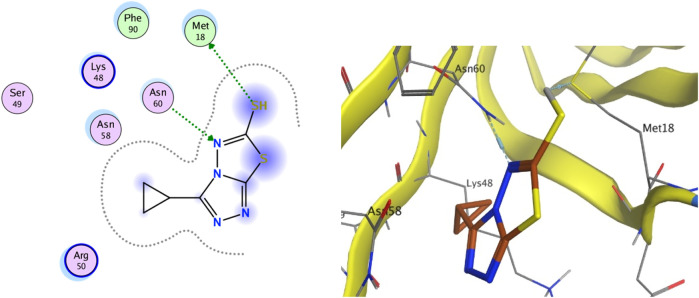
(Left) 2D ligands interaction diagram of the most active compounds **C2** with surrounding residues of the most probable binding site. (Right) 3D diagram of compound **C2** binding to this site.

The integral outer membrane protein X (OmpX) from *E. coli* belongs to a family of highly conserved bacterial proteins that promote bacterial adhesion to and entry into mammalian cells. Moreover, these proteins have a role in the resistance against attack by the human complement system (Hermansen et al., 2022; Vogt and Schulz, 1999). These proteins are highly conserved among pathogenicand non-pathogenic bacteria. For this binding site (1QJ8), both GlideScore and Model scores have a significant positive correlation with biological activity (Figure 9). The binding site binds well to **C2** and can form hydrogen bonding interactions. Compound **C2** has a strong interaction force with urinary methionine and aspartic acid. And the twist angle of **C2** has changed, which may be caused by newly formed hydrophobic interactions and van der Waals forces between **C2** and the ligand. This indicates that our compound **C2** may induce cell apoptosis by interacting with this protein.

## 3 Conclusion

In this paper, some triazole derivatives were prepared using carbon disulfide as starting material and mild reaction conditions. B-series compounds generally have stronger ability to remove metal ions than C-series compounds. The removal efficiency of C1-C5 for Pb^2+^, Cd^2+^, Ca^2+^ and Mg^2+^ in aqueous solution is 47%–67%, while the removal efficiency of B1-B5 for Pb^2+^, Cd^2+^, Ca^2+^ and Mg^2+^ in aqueous solution is 67%–87%. Overall, the metal removal effect is good. *In vitro* antibacterial activity evaluation values, **C2** has good antibacterial activity against *E. coli* MIC concentrations of 2 μg/mL. In addition, compound **C2** showed inhibitory activity against all *S*. *aureus* at a MIC of 32 μg/mL. Compound **C2** on drug resistance study suggested that **C2** has a low spontaneous frequency of resistance on *E. coli ATCC 25922, S. aureus ATCC 29213* and MASA. **C2** has low toxicity, no hemolysis within the safe range, and has a good biofilm inhibition effect. It also has a good anti-inflammatory effect. Next, we will carry out in-depth modification or coupling of other antimicrobial moieties such as antimicrobial peptides on the basis of **C2** with better antimicrobial ability. Since we found that **C2** has better antimicrobial activity for *E. coli* than *S. aureus*, we will test more negative bacteria with the drug. We will also analyse the targets and conduct research on the bactericidal mechanism based on network pharmacology. Finally, we will conduct some *in vivo* experiments to verify our efficacy.

## 4 Experimental section

### 4.1 Chemically synthetical experiments

All the key intermediates and final products were identified with ^1^H NMR and ^13^C NMR, recorded in a Bruker Avance 400 (^1^H at 400 MHz, ^13^C at 100 MHz), and chemical shifts were reported in parts per million using the residual solvent peaks as internal standards (CDCl_3_ = 7.26 ppm for ^1^H NMR and 77.16 ppm for ^13^C NMR). All compounds were characterized by MS (ESI).

#### 4.1.1 Hydrazinecarbothiohydrazide (A)

We stirred 30 mL of 80% hydrazine hydrate and 60 mL of water at 0°C for 15 min, then slowly added 9 mL of carbon disulfide to the system and refluxed for 10 h. After the reaction is completed, the crude product obtained after vacuum filtration is recrystallized and purified in hot water to obtain pure intermediate **A**. ^1^H NMR (400 MHz, DMSO) δ = 8.67 (s, 2H), 4.47 (s, 4H). ^13^C NMR (100 MHz, DMSO) δ = 181.99. TOF-MS, m/z: [M + H^+^], calcd. for CH_7_N_4_S^+^, 107.0313, found: 107.0357. Melting point 172°C.

#### 4.1.2 4-Amino-5-methyl-4H-1,2,4-triazole-3-thiol (B1)

Add intermediate **A** (10.0 mmol) to carboxylic acid (20.0 mmol) and reflux for 4 h. After the reaction is completed, pour it into a beaker while it is hot, cool it to room temperature, and then filter and collect the crude product under reduced pressure. Recrystallize from ethanol, precipitate solid through vacuum filtration, and collect pure intermediate **B1**. Yield, 56%. White solid powder. ^1^H NMR (400 MHz, DMSO) δ = 5.51 (s, 2H), 2.24 (s, 3H). ^13^C NMR (100 MHz, DMSO) δ = 165.80, 149.46, 10.85. TOF-MS, m/z: [M + H^+^], calcd. For C_3_H_7_N_4_S^+^, 131.0313, found: 131.0523. Melting point 138°C.

#### 4.1.3 4-Amino-5-cyclopropyl-4H-1,2,4-triazole-3-thiol (B2)

This compound was prepared as described in the general procedure for synthesizing compound **B2** using compound **B1**. Yield, 66%. White solid powder. ^1^H NMR (400 MHz, DMSO) δ = 13.30 (s, 1H), 5.54 (s, 2H), 2.06–1.99 (m, 1H), 1.00–0.84 (m, 4H). ^13^C NMR (100 MHz, DMSO) δ = 166.18, 153.95, 7.42, 5.18. TOF-MS, m/z: [M + H^+^], calcd. for C_5_H_9_N_4_S^+^, 157.2070, found: 157.2330. Melting point 146°C.

#### 4.1.4 4-Amino-4H-1,2,4-triazole-3-thiol (B3)

This compound was prepared as described in the general procedure for synthesizing compound **B3** using compound **B1**. Yield, 70%. White solid powder. ^1^H NMR (400 MHz, DMSO) δ = 13.60 (s, 1H), 8.41 (s, 1H), 5.64 (s, 3H). ^13^C NMR (100 MHz, DMSO) δ = 166.02, 142.36. TOF-MS, m/z: [M + H^+^], calcd. for C_2_H_5_N_4_S^+^, 117.1420, found: 117.1530. Melting point 121°C.

#### 4.1.5 4-Amino-5-(methoxymethyl)-4H-1,2,4-triazole-3-thiol (B4)

This compound was prepared as described in the general procedure for synthesizing compound **B4** using compound **B1**. Yield, 66%. White solid powder. ^1^H NMR (400 MHz, DMSO) δ = 13.66 (s, 1H), 5.53 (s, 2H), 4.38 (s, 2H), 3.28 (s, 2H). ^13^C NMR (100 MHz, DMSO) δ = 166.67, 149.11, 63.03, 58.32. TOF-MS, m/z: [M + H^+^], calcd. for C_4_H_9_N_4_OS^+^, 161.1950, found: 161.1943. Melting point 156°C.

#### 4.1.6 4-Amino-5-ethyl-4H-1,2,4-triazole-3-thiol (B5)

This compound was prepared as described in the general procedure for synthesizing compound **B5** using compound **B1**. Yield, 52%. White solid powder. ^1^H NMR (400 MHz, DMSO) δ = 13.37 (s, 1H), 5.47 (s, 2H), 2.63–2.57 (m, 2H), 1.17–1.53 (m, 3H). ^13^C NMR (100 MHz, DMSO) δ = 166.15, 153.58, 18.40, 10.71. TOF-MS, m/z: [M + H^+^], calcd. for C_4_H_9_N_4_S^+^, 145.1960, found: 145.1977. Melting point 143°C.

#### 4.1.7 3-Methyl-[1,2,4]triazolo [3,4-*b*][1,3,4]thiadiazole-6-thiol (C1)

We weigh intermediate **B1** (10.0 mmol) and add it to a 30 mL methanol solution containing potassium hydroxide (12.0 mmol), stirring at room temperature for 10 min. Add carbon disulfide (40.0 mmol) in batches under stirring conditions, and reflux for 20 h after dripping. After complete reaction (TLC monitoring, Petroleum ether:Ethyl acetate = 3:1), the system was poured into ice water and adjusted to pH 3-4 with dilute hydrochloric acid. Reduce pressure filtration, wash the precipitate with cold ethanol, dry and collect intermediate **C1**. Compound **C1** is a white solid. Yield, 46%. ^1^H NMR (400 MHz, DMSO) δ = 2.59 (s, 3H). ^13^C NMR (100 MHz, DMSO) δ = 153.96, 142.18, 10.06. TOF-MS, m/z: [M + H^+^], calcd. for C_4_H_5_N_4_S_2_^+^, 173.2240, found: 173.2251. Melting point 133°C.

#### 4.1.8 3-Cyclopropyl-[1,2,4]triazolo [3,4-*b*][1,3,4]thiadiazole-6-thiol (C2)

This compound was prepared as described in the general procedure for synthesizing compound **C2** using compound **C1**. Compound **C2** is a white solid. Yield, 50%. ^1^H NMR (400 MHz, DMSO) δ = 2.33–2.26 (m, 1H), 1.26–1.18 (m, 4H). ^13^C NMR (100 MHz, DMSO) δ = 154.27, 146.08, 7.70, 5.81. TOF-MS, m/z: [M + H^+^], calcd. for C_6_H_7_N_4_S_2_^+^, 199.2620, found: 199.2653. Melting point 143°C.

#### 4.1.9 [1,2,4]triazolo [3,4-*b*][1,3,4]thiadiazole-6-thiol (C3)

This compound was prepared as described in the general procedure for synthesizing compound **C3** using compound **C1**. Compound **C3** is a white solid. Yield, 58%. ^1^H NMR (400 MHz, DMSO) δ = 9.68 (s, 1H),8.44 (s, 1H). ^13^C NMR (100 MHz, DMSO) δ = 154.64. TOF-MS, m/z: [M + H^+^], calcd. for C_3_H_3_N_4_S_2_^+^, 159.1970, found: 159.1993. Melting point 112°C.

#### 4.1.10 3-(methoxymethyl)-[1,2,4]triazolo [3,4-*b*][1,3,4]thiadiazole-6-thiol (C4)

This compound was prepared as described in the general procedure for synthesizing compound **C4** using compound **C1**. Compound **C4** is a white solid. Yield, 66%. ^1^H NMR (400 MHz, DMSO) δ = 4.75 (s, 2H), 3.33 (s, 3H). ^13^C NMR (100 MHz, DMSO) δ = 154.64, 143.75, 124.63, 63.17, 58.80. TOF-MS, m/z: [M + H^+^], calcd. for C_5_H_7_N_4_OS_2_^+^, 203.2500, found: 203.2556. Melting point 139°C.

#### 4.1.11 3-Ethyl-[1,2,4]triazolo [3,4-*b*][1,3,4]thiadiazole-6-thiol (C5)

This compound was prepared as described in the general procedure for synthesizing compound **C5** using compound **C1**. Compound **C5** is a white solid. Yield, 51%. ^1^H NMR (400 MHz, DMSO) δ = 3.02–2.98 (q, 3H), 1.35–1.31 (t, 4H). ^13^C NMR (100 MHz, DMSO) δ = 154.04, 146.27, 18.17, 10.35. TOF-MS, m/z: [M + H^+^], calcd. for C_5_H_7_N_4_S_2_^+^, 187.2510, found: 187.2543. Melting point 135°C.

### 4.2 The antibacterial activity of the compounds

#### 4.2.1 Determination of minimum inhibitory concentration

The minimum inhibitory concentrations (MICs) of the test compounds were determined using a broth microdilution method according to the Clinical and Laboratory Standards Institute (CLSI) guidelines (Pierce et al., 2023). A single colony of test *S. aureus* strain was inoculated from TSB agar plate to 5 mL Mueller Hinton broth (MH) and then overnight incubated at 37°C. Cells were diluted to a final concentration of approximately *Escherichia coli ATCC25922* and *S. aureus* cells (∼10^5^ CFU/mL, CFU equals colony forming units) in a 96-well microtiter plate. The compounds were then added at a series concentration (0.25, 0.5, 1, 2, 4, 8, 16, 32, 64, 128, 256 μg/mL) and the plate was incubated at 37°C for 18 h. The MICs are determined as the minimum concentration of the visually clear wells. Three independent assays were performed for all the tests.

#### 4.2.2 Time-killing kinetics

For time-killing kinetic experiments, *E*. *coli* ATCC25922 and *S*. *aureus* ATCC 29213 cells (10^5^ CFU/mL) were exposed to 2 × MIC to 8 × MIC. As an untreated control, the bacteria were incubated with DMSO instead of **C2**. At each time interval after treatment over 22 h, Aliquots of 100 µL were taken at different times and appropriately diluted in PBS buffer (pH 7.4), and spread onto LB plates. The number of CFU were counted after incubating at 37°C overnight. Three independent experiments were conducted. Each experiment was performed in triplicate.

#### 4.2.3 Drug resistance study

The drug resistance study of compound **C2** was performed by following the protocol of previous study (Xiao et al., 2024). The initial MIC values of **C2** against *E*. *coli ATCC25922* and *S. aureus ATCC29213* were determined according to method described above. The bacteria in the 0.5 × MIC wells from the above experiment were diluted to approximately 1 × 10^5^ CFU mL^-1^ with MH for the next MIC test. Various concentrations of compounds were added to the corresponding bacteria, the MICs are determined after incubated 24 h at 37°C. The process was repeated continuously for 28 days, assays were carried out with biological replicates.

### 4.3 The toxicity of the compounds

#### 4.3.1 Hemolysis assay

The hemolysis assay conducted following a previously reported procedure, with minor modifications (Ajish et al., 2023). One hundred microliters of **C2** at different concentrations in PBS (Phosphate-Buffered Saline) was mixed with 100 µL PBS containing 4% defibrinated rabbit erythrocytes (Nanjing Maojie Microbial Technology Co., Ltd., Nanjing, China), resulting in a final concentration of 4, 8, or 16 μg/mL, respectively. To establish a positive control, 1% Triton X-100 was used, whereas sterile PBS served as the negative control. Following incubation at 37°C for 1 h, erythrocytes were separated from the supernatant by centrifugation at 1,000 g for 5 min. Subsequently, the absorbance at 490 nm was recorded, and the hemolysis ratio was calculated using the following formula: hemolysis (%) = (sample − PBS)/(Triton − PBS) × 100%. The experiments were repeated thrice.

#### 4.3.2 Cytotoxicity assay

Cell viability was assessed using the cell counting kit 8 (Beyotime, Shanghai, China) method. Cytotoxicity assay was done as described before with minor modifications (Xiao et al., 2024). Briefly, VERO cells were seeded at a density of 103 cells/well in DMEM in a 96-well plate. After 24 h of incubation in a cell incubator, various concentrations of **C2** (1–256 μg/mL) were added to the cells. Wells that contain 0.3% DMSO and did not contain **C2** were used as the control. After incubation for 24 h, wells were carefully washed twice with sterile PBS. Next, 90 µL DMEM was added to each well, and 10 µL of CCK8 reagent was introduced in a dark environment. After 2 h of incubation at 37°C, OD_450_ was recorded using a microplate reader. The results were calculated as follows: Cell viability (%) = (OD_450_ sample value − OD_450_ blank hole)/(OD_450_ value of untreated control − OD_450_ blank hole) × 100%.

### 4.4 Biofilm formation assay

The biofilm was quantitatively analyzed by the crystal violet method (Yap et al., 2023). *S. aureus* ATCC 29213 was incubated in test tubes with TSB (5 mL) at 37°C for 24 h. After that, the *S. aureus* ATCC 29213 was diluted 100-fold with fresh TSB medium containing 1% w/v glucose, then the diluted bacterial solution and the compound solution were added to a 96-well plate filled with TSB (200 μL) with 1% w/v glucose. **C2** were directly added to the wells to reach concentrations ranging from 256 to 1 μg/mL to assess the concentration at which inhibition of biofilm formation. The control group was added to the same amount of DMSO per well. *S. aureus* ATCC 29213 culture was removed and washed three times with PBS and dried; 0.1% crystal violet solution was then added to each well for 15 min staining. After the excess crystal violet solution was removed and washed three times with PBS, the pigment was dissolved in 95% ethanol. Finally, the absorbance value was measured at 595 nm by a microplate reader. The formula for calculating the biofilm inhibition rate is: OD_595_control − OD_595_/OD_595_ control × 100%.

The minimum concentration of the tested compounds required to eradicate the preformed biofilm. In this assay, the *S. aureus* ATCC 29213 biofilms were formed prior to the addition of treatment compounds. First, *S. aureus* ATCC 29213 suspension in TSB supplemented with 1% v/v glucose was added to each well of a 96-well microtiter plate and incubated for 24 h at 37°C. Next, sub-MIC concentration values of compounds **C2** were directly added to the wells to reach concentrations ranging from 256 to 1 μg/mL to assess the concentration at which the percentage of eradicate of biofilm formation. The other steps are similar to the inhibition experiment.

### 4.5 The anti-inflammatory activity of the compounds

In LPS stimulated RAW 264.7 cells were studied according to the method (El-Shahid et al., 2021). All cells were treated with the studied compound and LPS or LPS alone for 24 h. To determine the level of NO production, nitrite accumulation was used as an indicator of NO production using a microplate assay based on the Griess reaction. Calculation The NO level of each of the tested cell supernatants was expressed as NO level of the tested cell supernatant × 100/NO level of the control.

### 4.6 Adsorption studies

Efficiency of the synthesized compounds towards the metal and heavy metal ions removal was evaluated using batch mode (Abo Markeb et al., 2017; Markeb et al., 2016; Hozien et al., 2020). The removal percentage (Removal, %), were calculated as presented in Equation: Removal,% = [(C_0_-C_e_)/C_0_] × 100. See Supplementary Appendix for more details.

### 4.7 Statistical analysis

The above experimental data is the average ± SEM (Standard Error of Mean) independent experiment of at least three data points. SPSS 22.0 software was used to analyze the data, and one-way analysis of variance (ANOVA) was used to process the statistical differences between the two groups.

### 4.8 Docking study

Selection of previously described 1QJ8 targets for molecular docking with **C2**. See Supplementary Appendix for more details.

## Data Availability

The original contributions presented in the study are included in the article/Supplementary Material, further inquiries can be directed to the corresponding author.
