# Supplementary material for: Synthesis of new triazole derivatives and their potential applications for removal of heavy metals from aqueous solution and antibacterial activities
Source: Front Chem. 2024 Oct 23;12:1473097. doi: 10.3389/fchem.2024.1473097 (PMC11537928; doi:10.3389/fchem.2024.1473097)
Supplement: Supplementary file 1 [file DataSheet1.doc]

**Synthesis of new triazole derivatives and their potential applications for removal of heavy metals from aqueous solution and antibacterial activities**

Chunyun Xu[[1]](#footnote-2)*, Na Yang^1^ , Haichun Yu^1^ , Xiaojing Wang^1^

*^1^Department of Dermatology, Maternity and Child Health Hospital of Qinhuangdao, Qinhuangdao, China, 066000, Qinhuangdao, China*

**Table of Contents**

**Adsorption studies**································································································1

**Docking study**···································································································2

**Spectral data**·····································································································3-13

**Adsorption studies**

The adsorption behavior of the compounds under investigation towards heavy metals (Pb^2+^ and Cd^2+^), and metal ions (Ca^2+^ and Mg^2+^) was carried out by batch adsorption experiments. A dose of adsorbent (W) (g/L) was added into a 100 mL conical flask containing heavy metal or metal ion solution with an initial concentration (C_0_). Typically, 25 mg of each synthesized compounds as adsorbent was added into the conical flask which contains 25 mL of the metal/ heavy metal ion solution (100 mg/L) as initial concentration. Depending on the experiment, pH of the solution was adjusted to 6.0 using NaOH or HCl, both at 0.1 M. The flask was shaken (180 rpm) at 25℃ using an incubating shaker and the mixture was left for 24 h to achieve the equilibrium. Next, the adsorbent was separated from the medium by filteration using 0.45 µm Nylon membrane filter. Then, the residual concentration of Pb^2+^, Cd^2+^, Ca^2+^ and Mg^2+^ in the solution, C_e_, was determined by Atomic Absorption Spectroscopy after digestion in HNO_3_ (10 %) overnight. UV/VIS spectrophotometer was used to confirm the equilbrium concentration of Pb^2+^ and Cd^2+^ in a form of complex that is extracted with chloroform after the reaction and lead and cadmium with dithizone.

**Docking study**

Selection of previously described 1QJ8 targets for molecular docking with C2. The files of sdf. structure of C2 active ingredient compounds were downloaded from PubChem database, and imported into ChemBio3D 14.0 software to adjust the spatial conformation of active ingredients, calculate the optimization of energy, and save in mol2 format. After AutoDockTools processing, the files were saved in pdbqt format. The three-dimensional crystal structure of the target protein was downloaded from the PDB protein database (https://www.rcsb.org/). The PDBID was 1QJ8. The water molecule and organic matter in the target protein were removed by Notedad2, and then the target protein was imported into AutoDockTools for hydrogenation, charge distribution, and atomic type addition. The pdbqt format file was saved. AutoDockVina was used for molecular docking, and the docking results were plotted with Pymol.

**Spectral data**


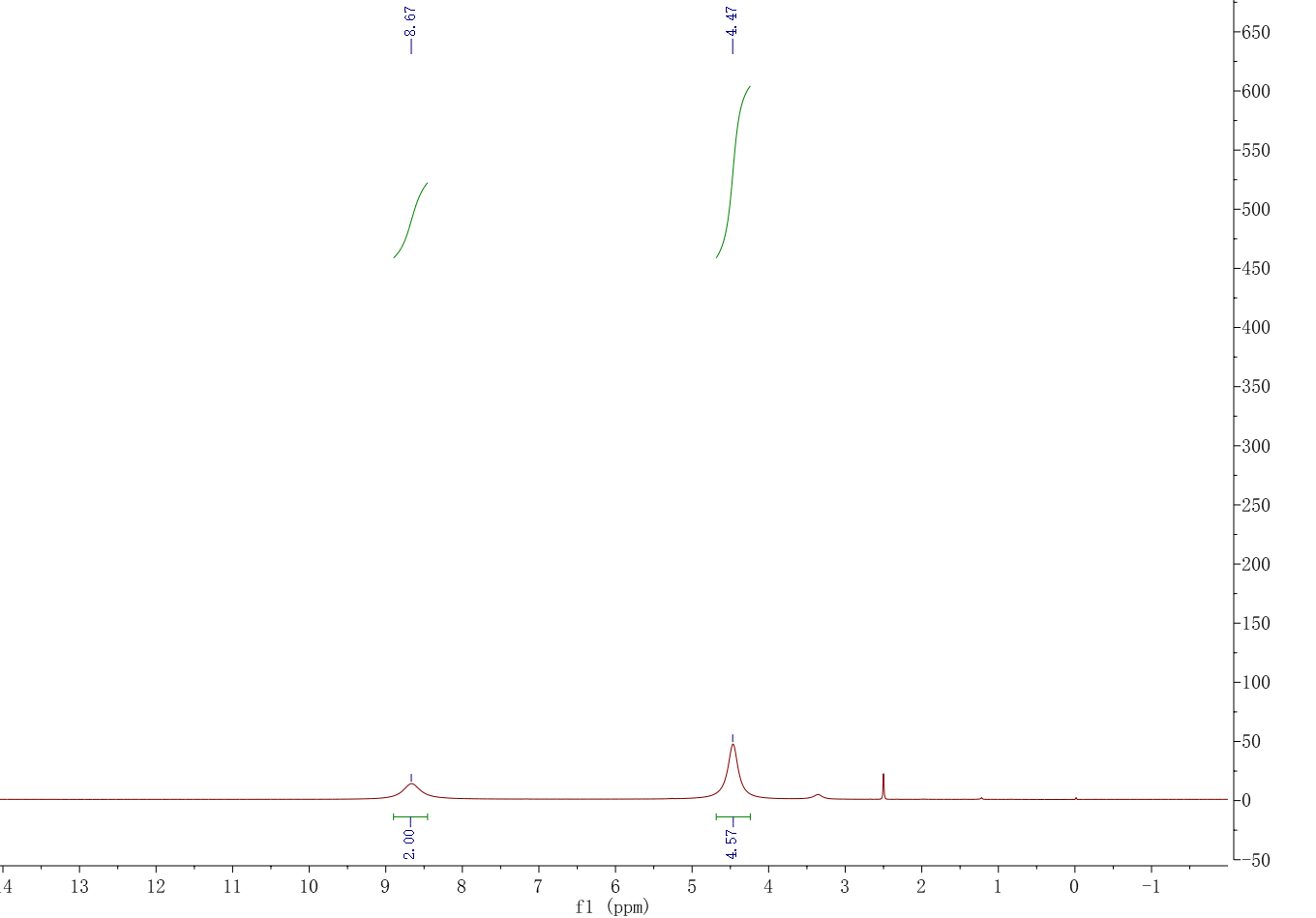


Fig. *^1^H NMR of* **A** (400 MHz, DMSO)


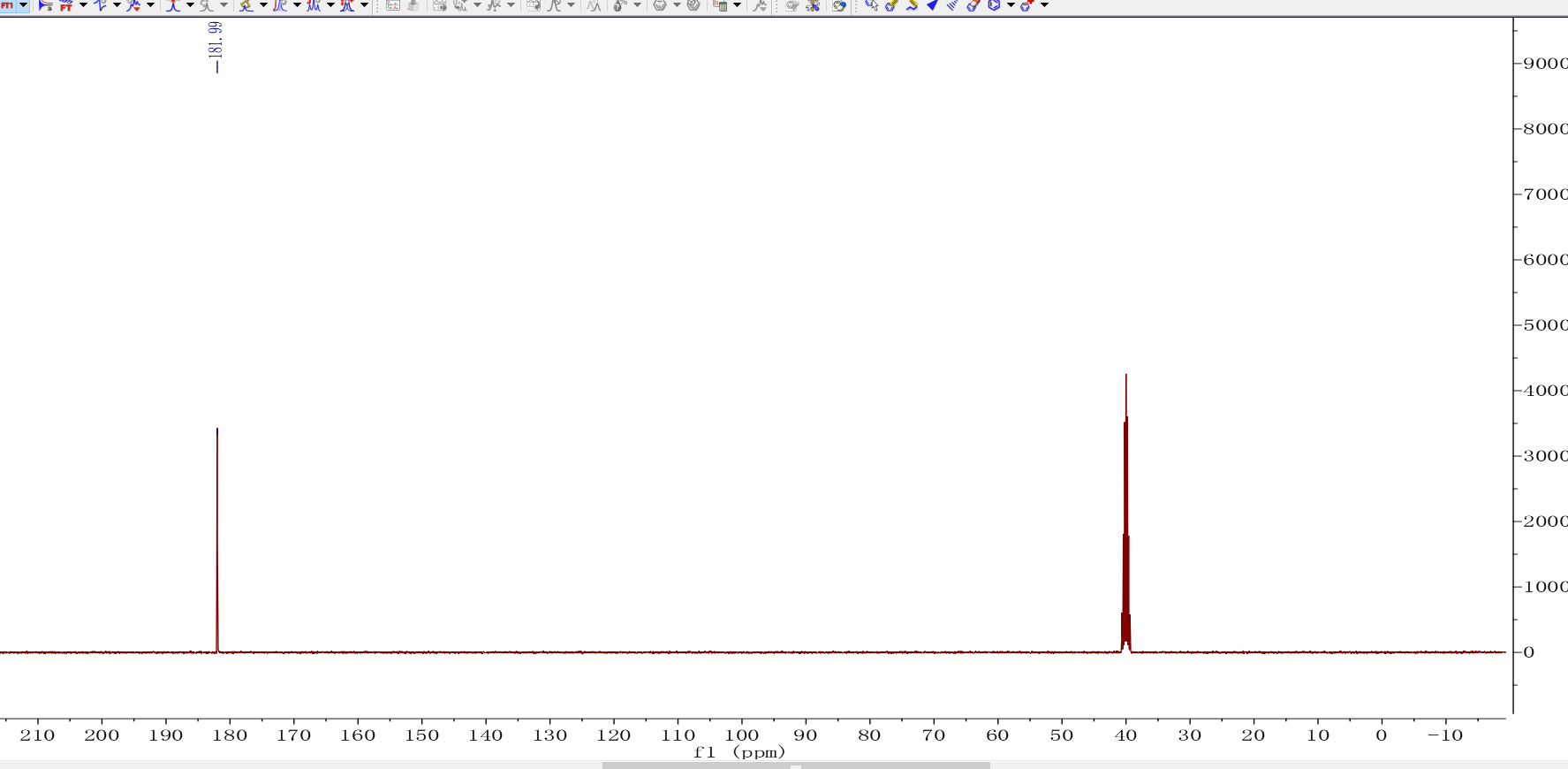


Fig. *^13^C NMR of* **A** (100 MHz, DMSO)


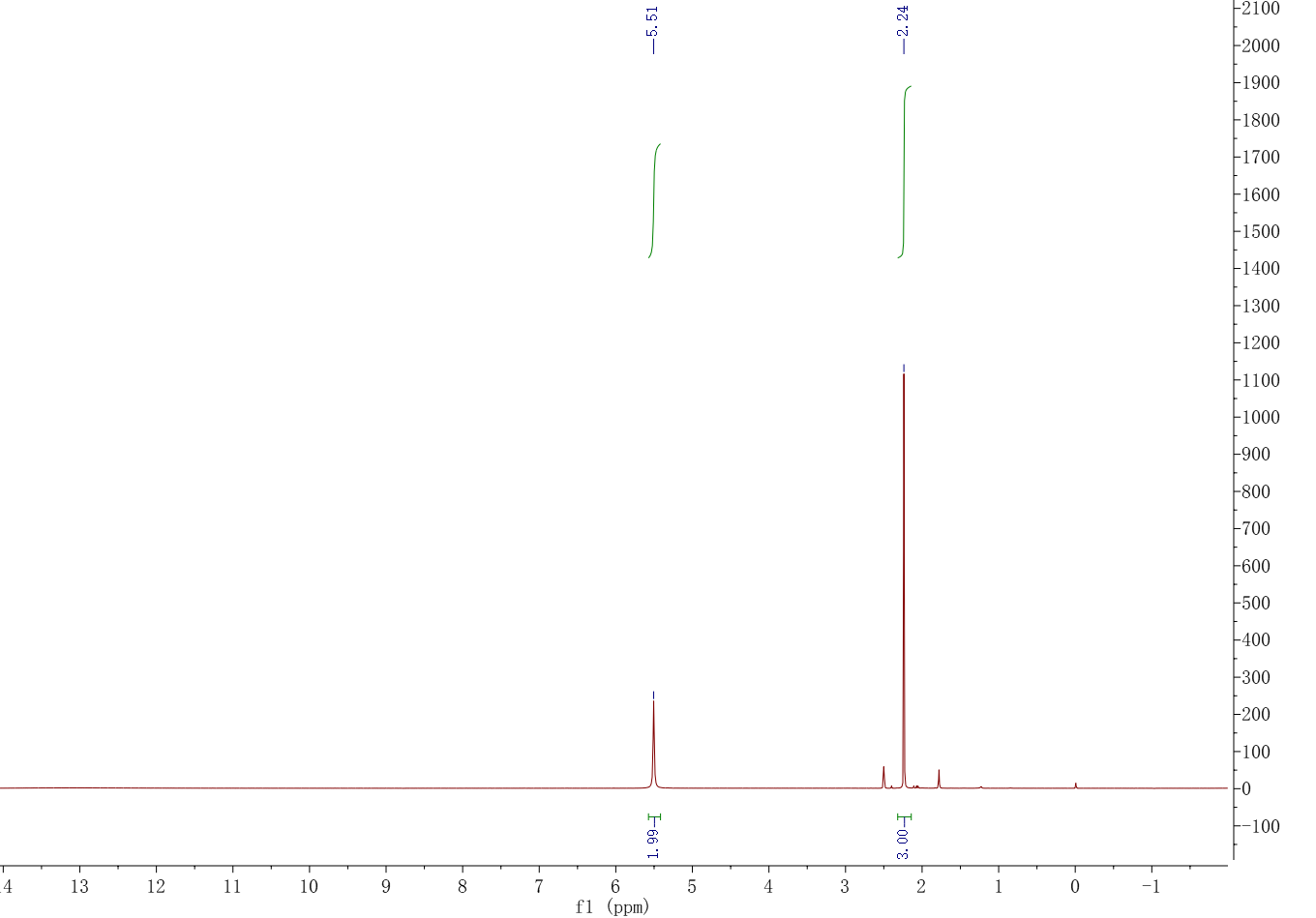


Fig. *^1^H NMR of* **B1** (400 MHz, DMSO)


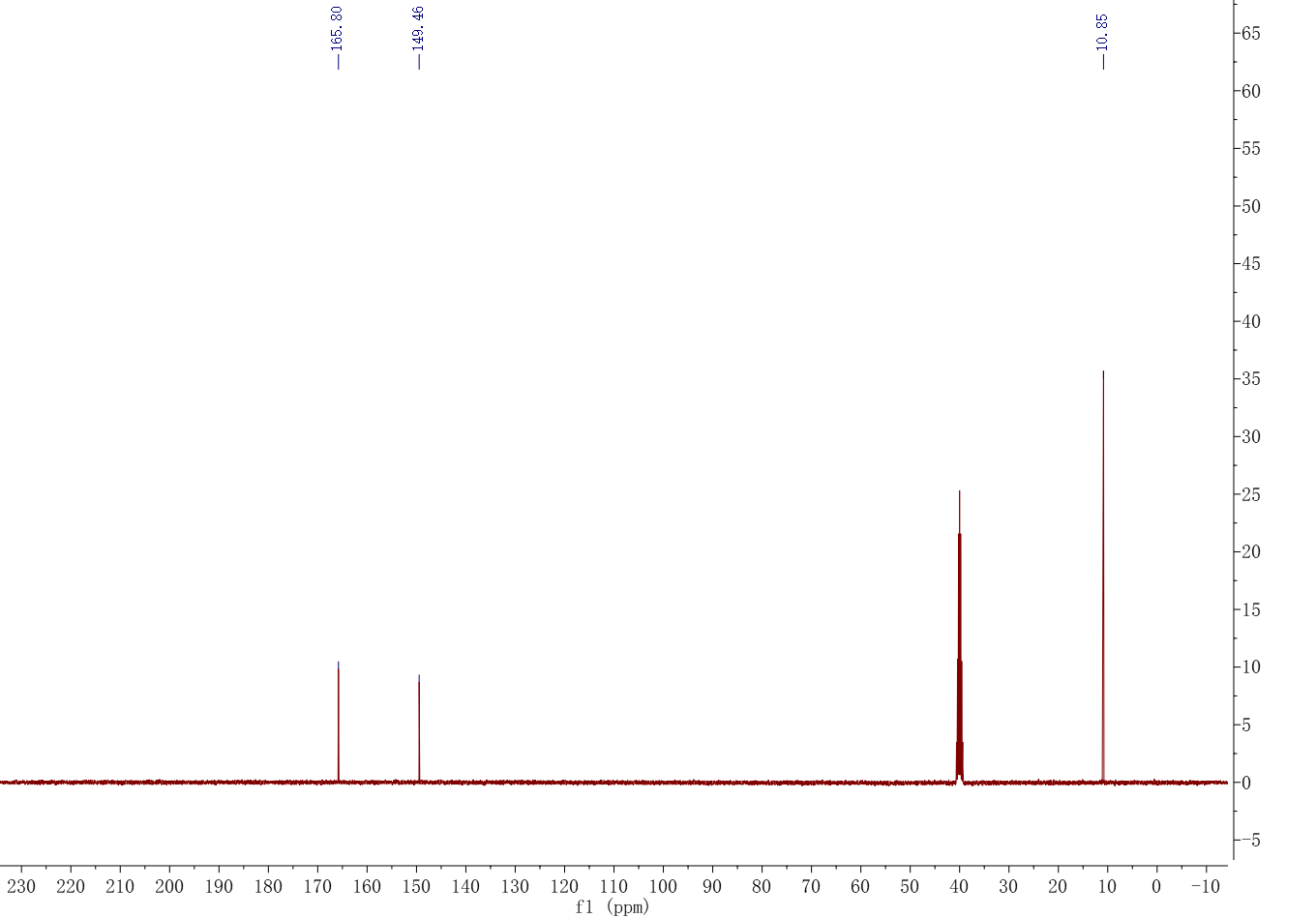


Fig. *^13^C NMR of* **B1** (100 MHz, DMSO)


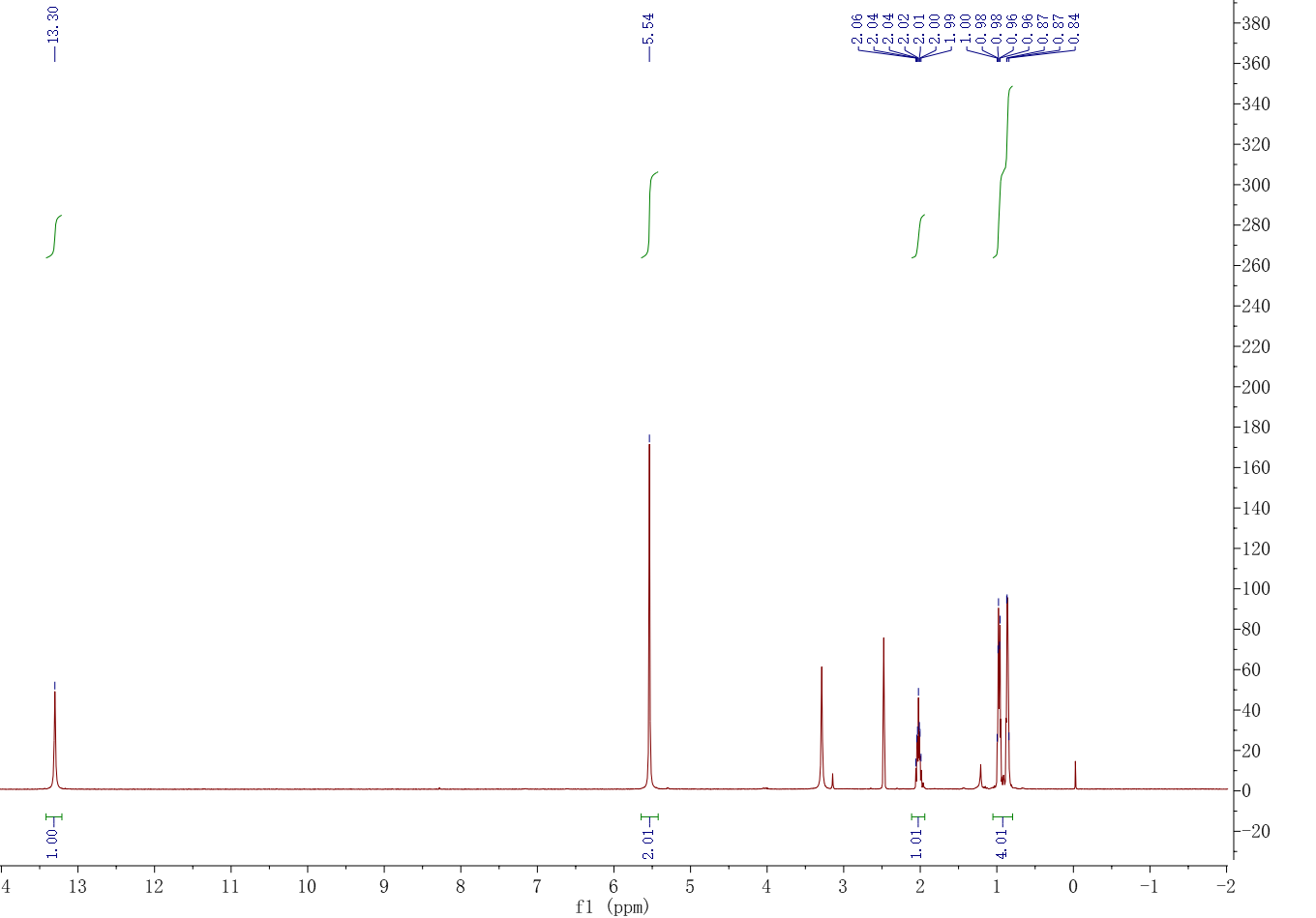


Fig. *^1^H NMR of* **B2** (400 MHz, DMSO)


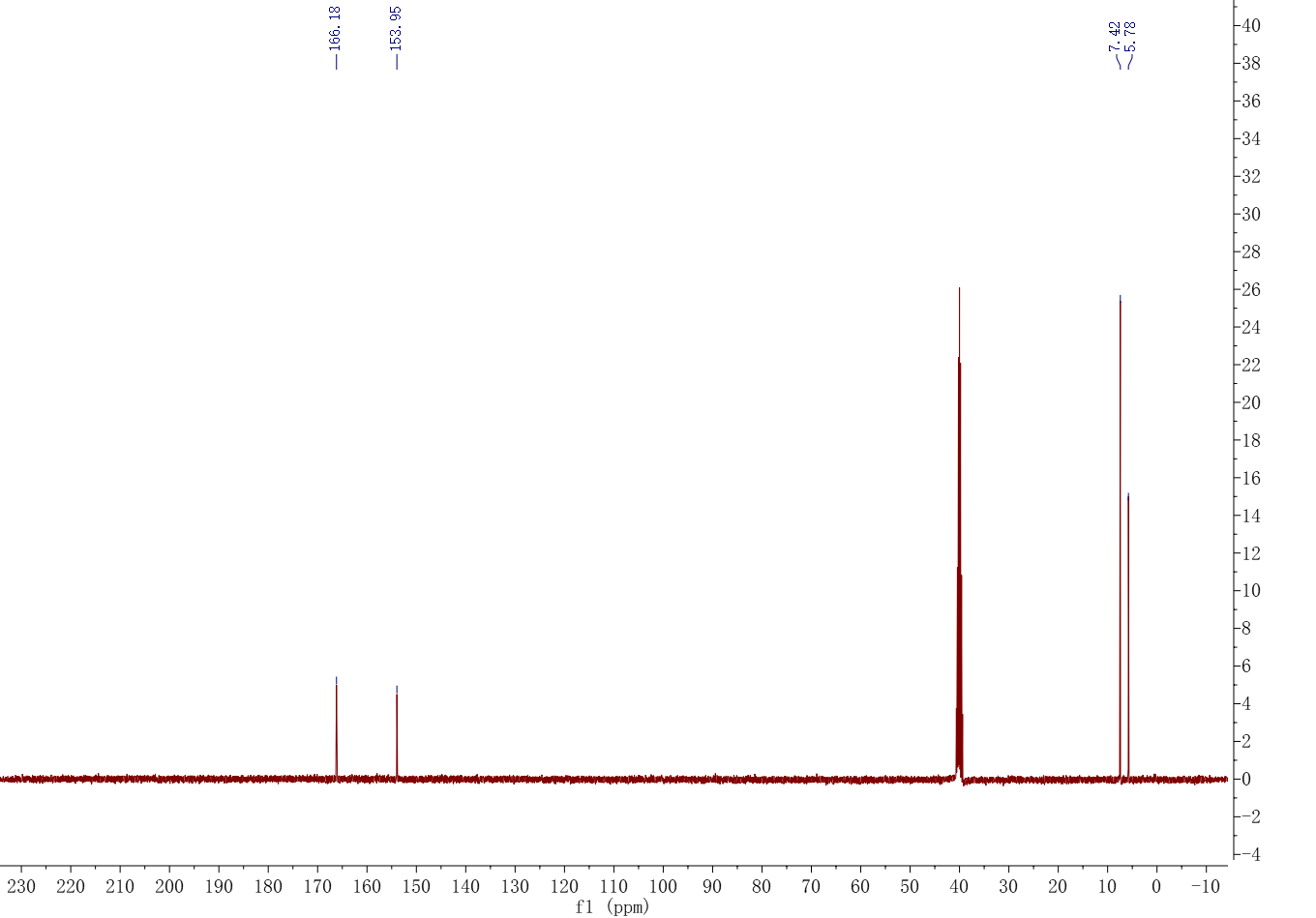


Fig. *^13^C NMR of* **B2** (100 MHz, DMSO)


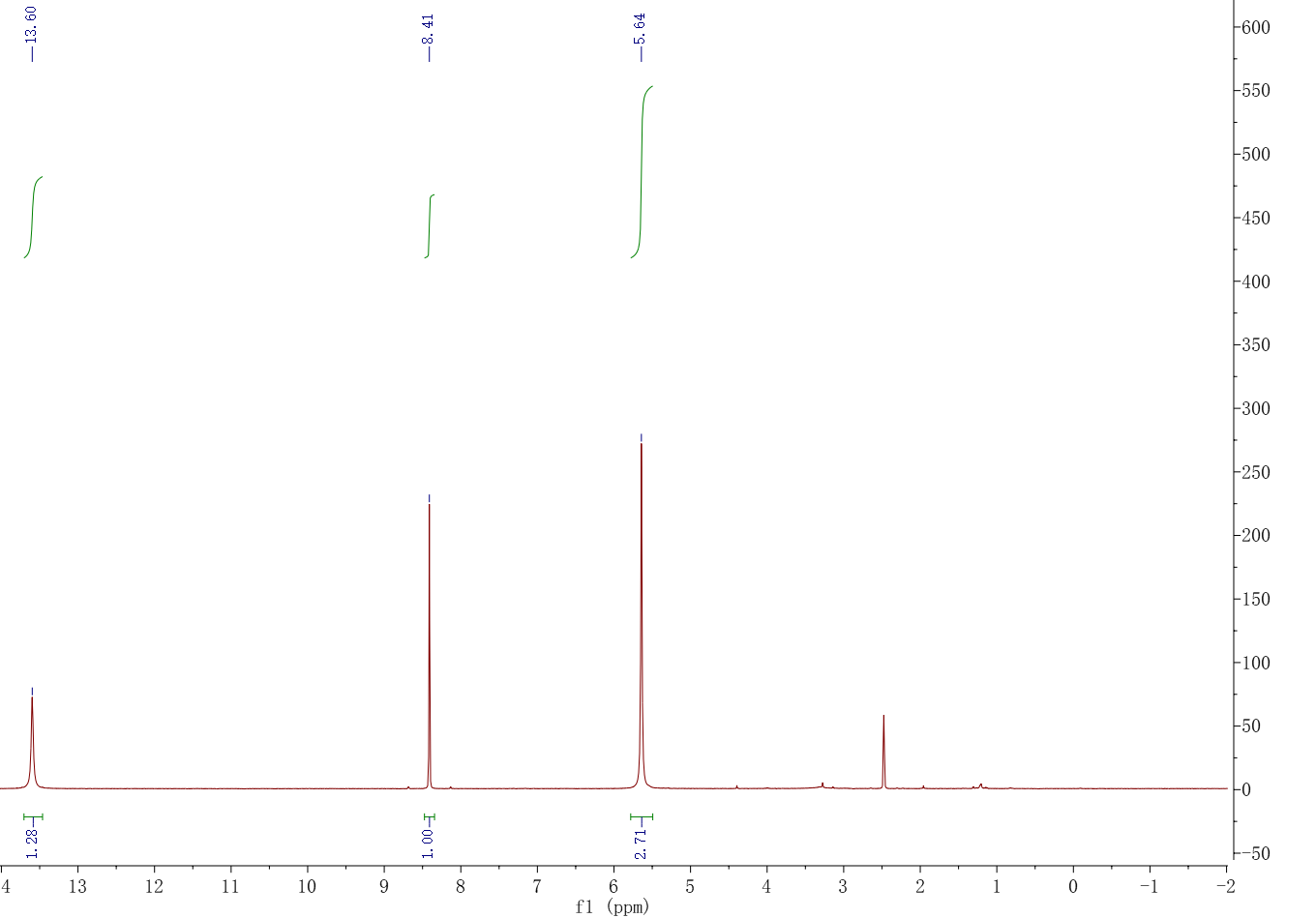


Fig. *^1^H NMR of* **B3** (400 MHz, DMSO)


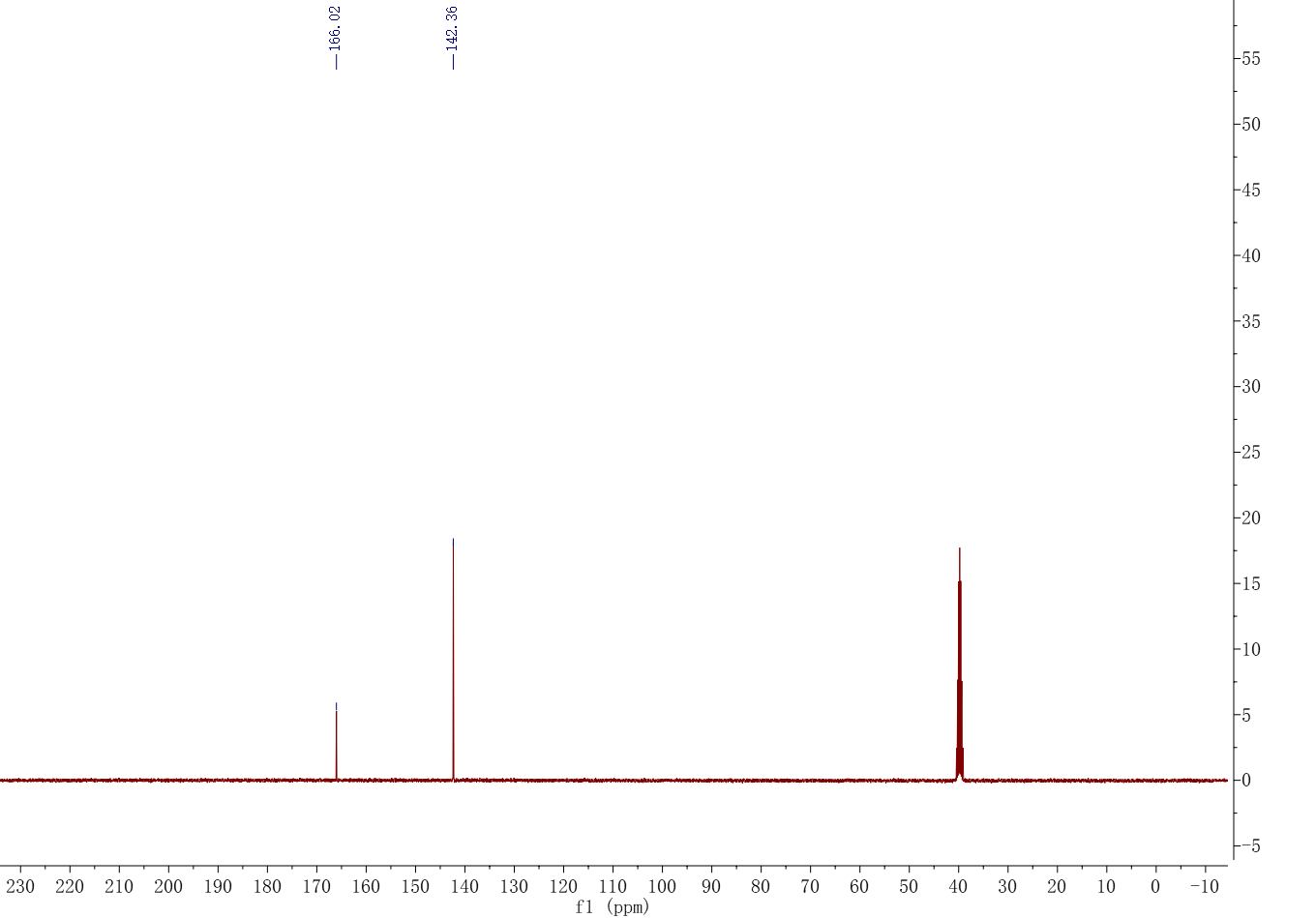


Fig. *^13^C NMR of* **B3** (100 MHz, DMSO)


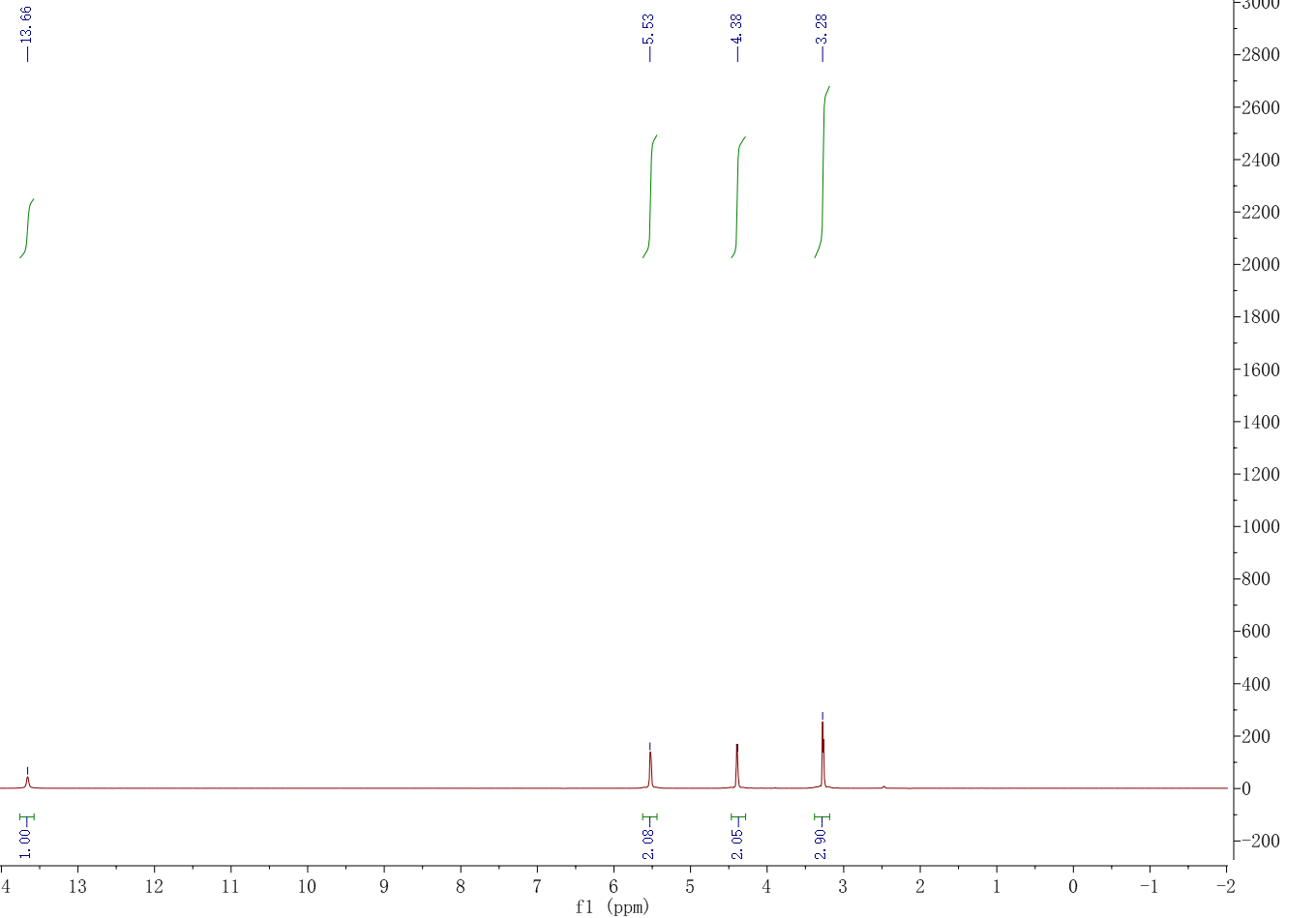


Fig. *^1^H NMR of* **B4** (400 MHz, DMSO)


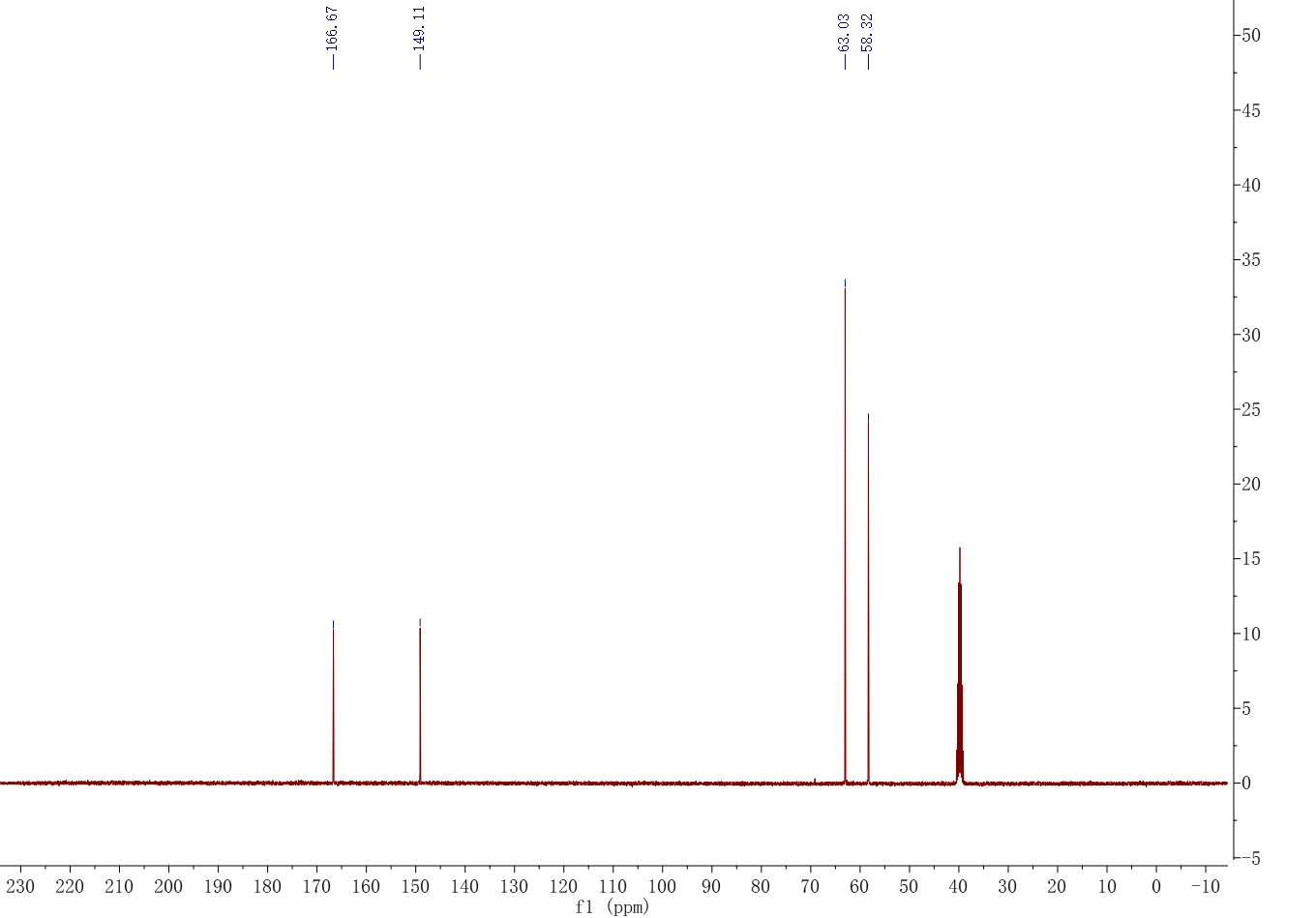


Fig. *^13^C NMR of* **B4** (100 MHz, DMSO)


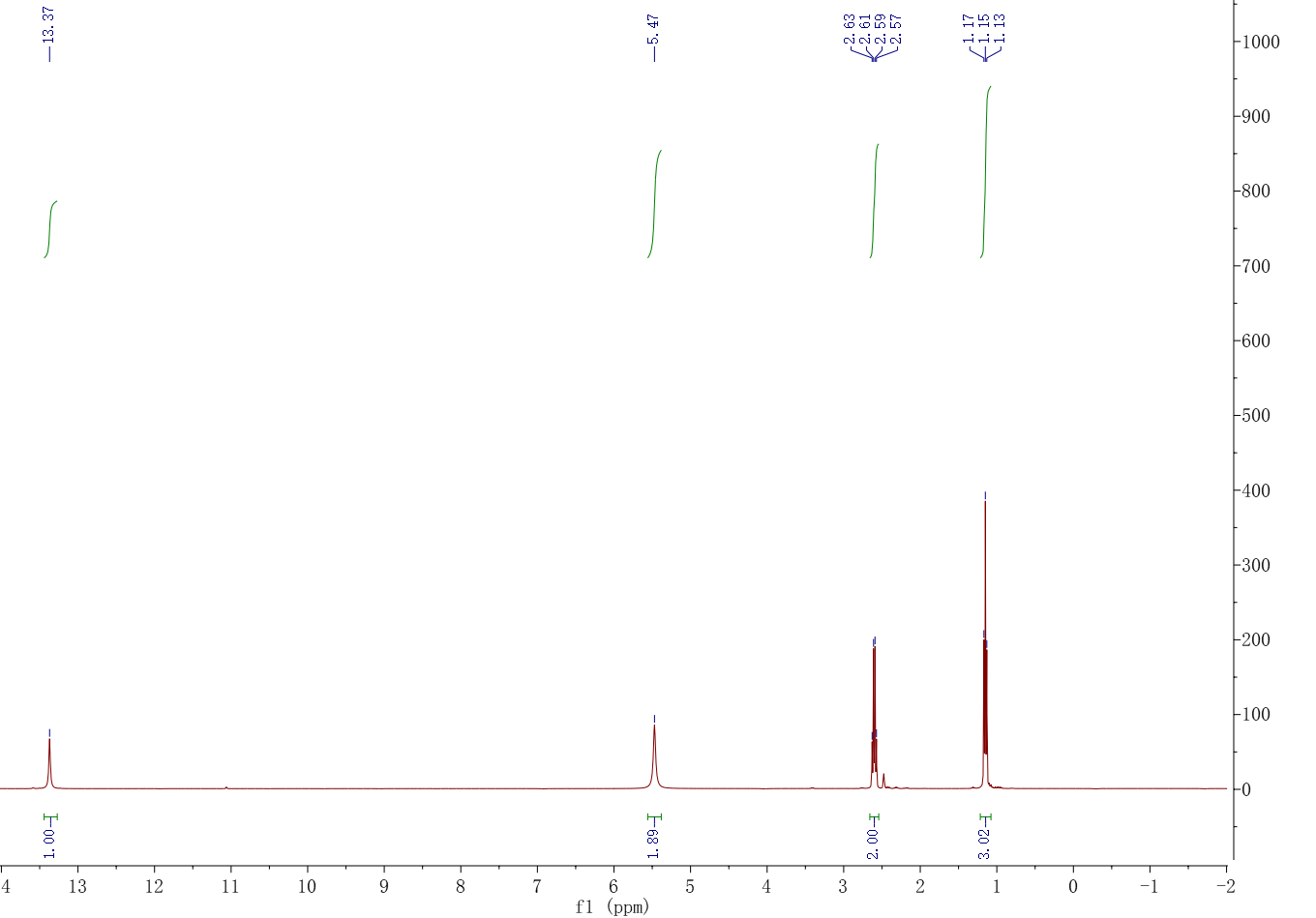


Fig. *^1^H NMR of* **B5** (400 MHz, DMSO)


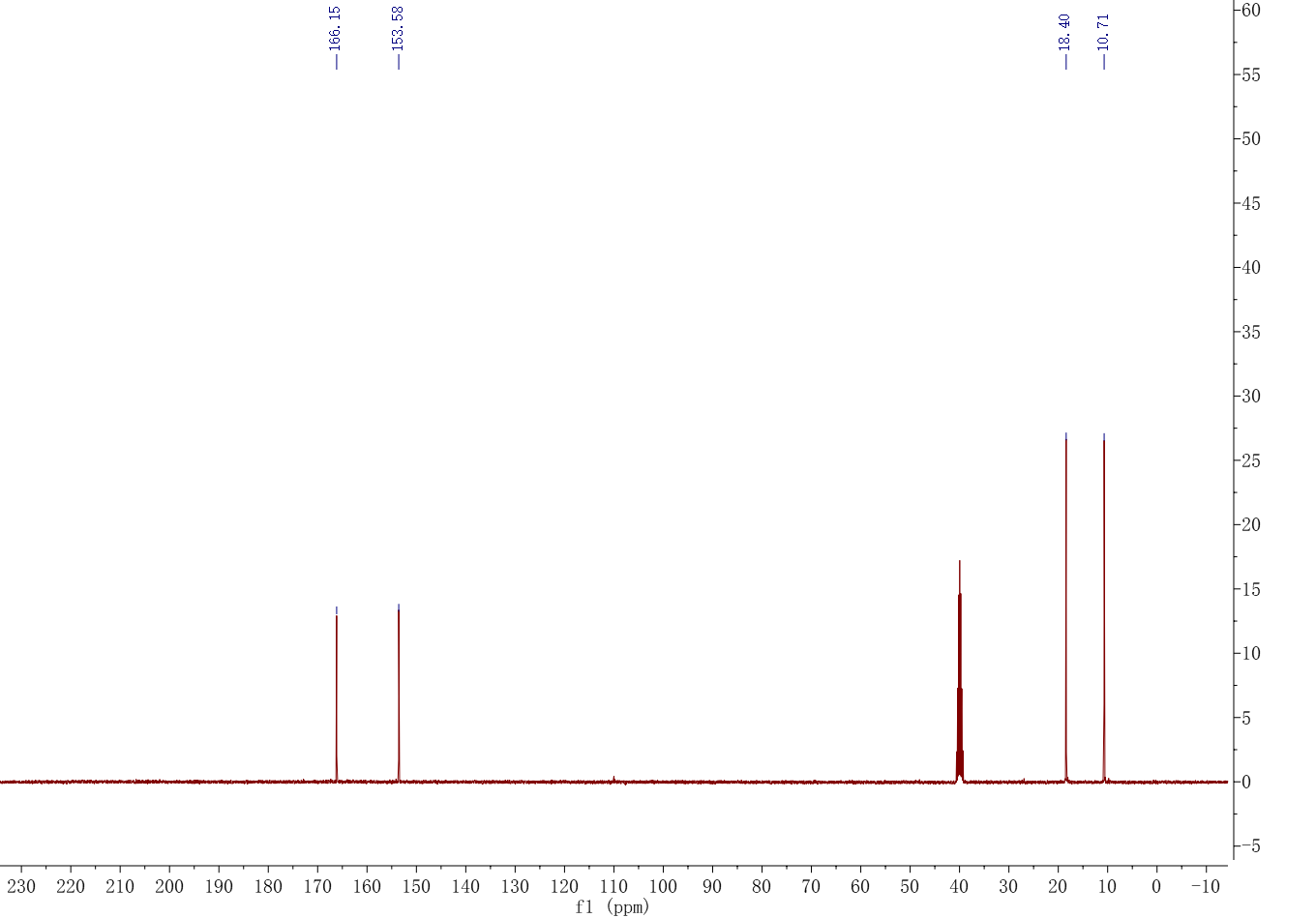


Fig. *^13^C NMR of* **B5** (100 MHz, DMSO)


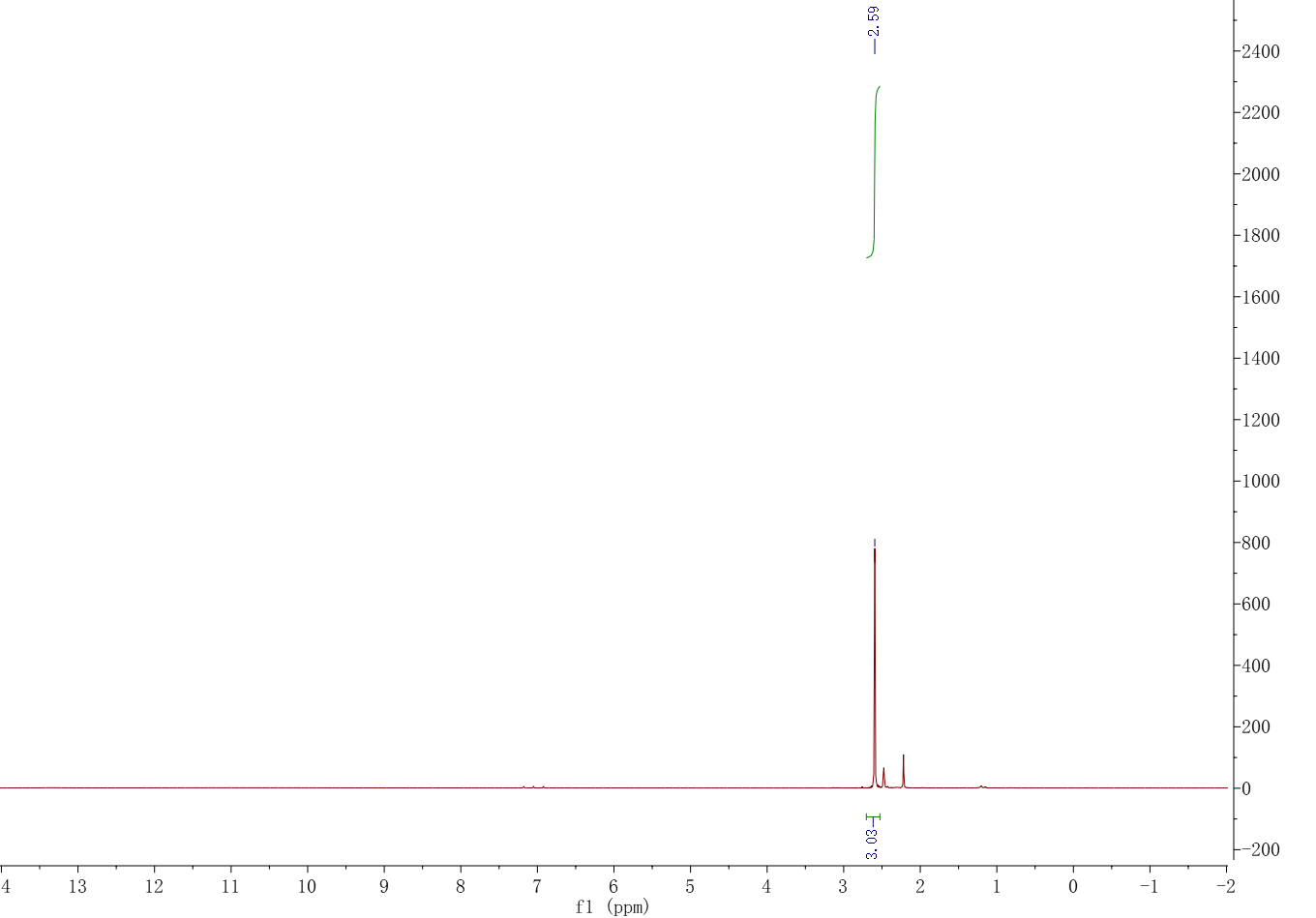


Fig. *^1^H NMR of* **C1** (400 MHz, DMSO)


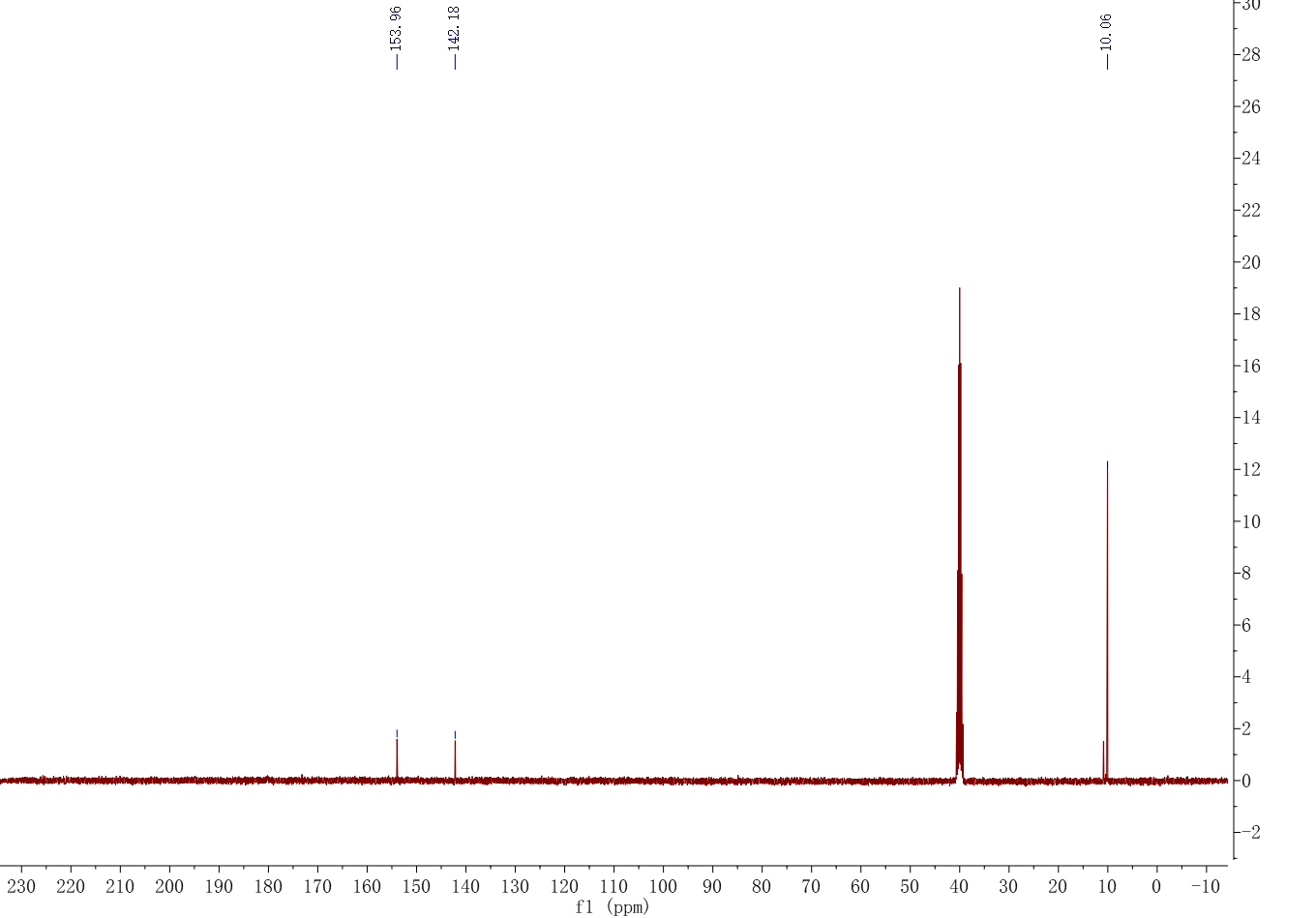


Fig. *^13^C NMR of* **C1** (100 MHz, DMSO)


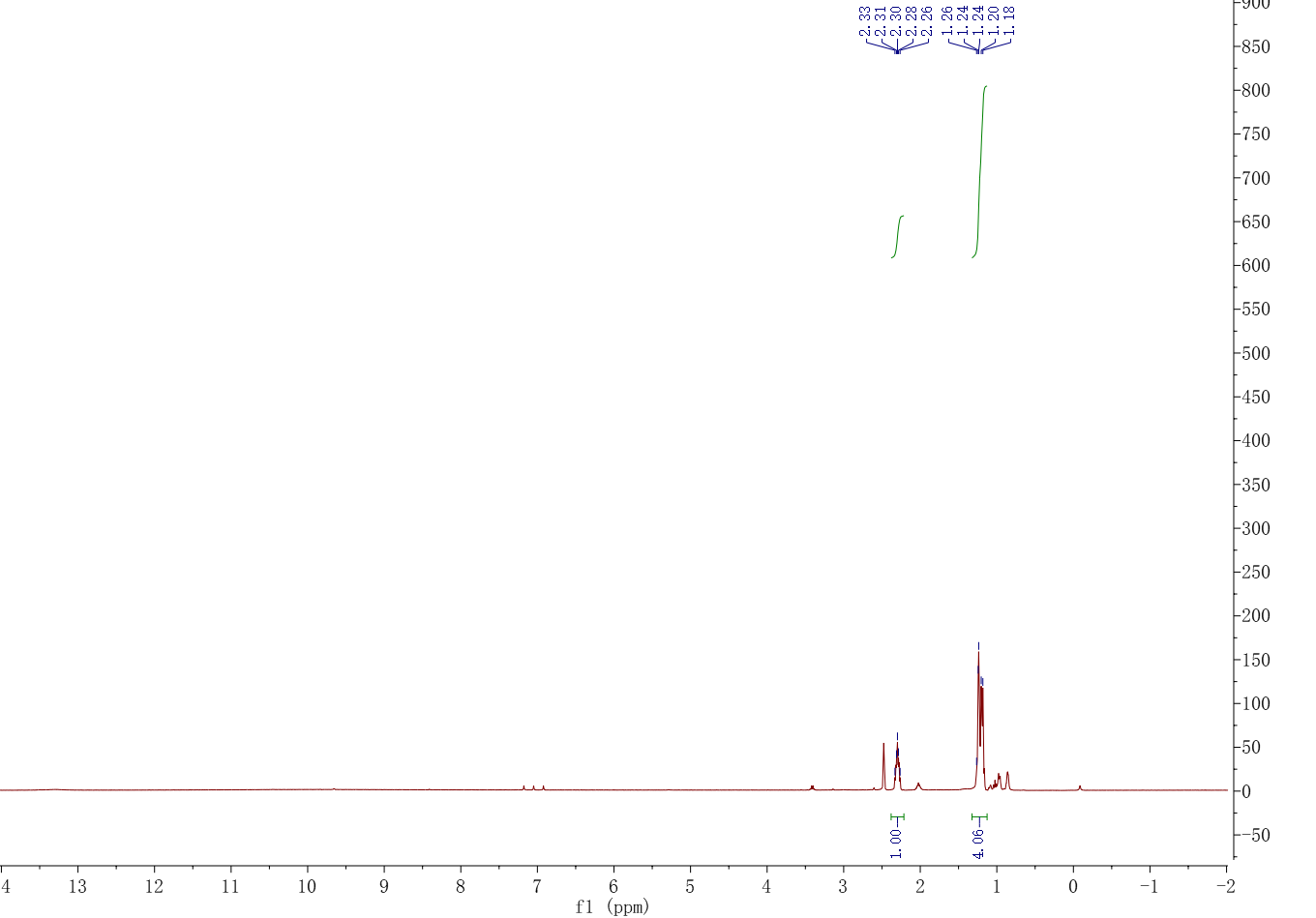


Fig. *^1^H NMR of* **C2** (400 MHz, DMSO)


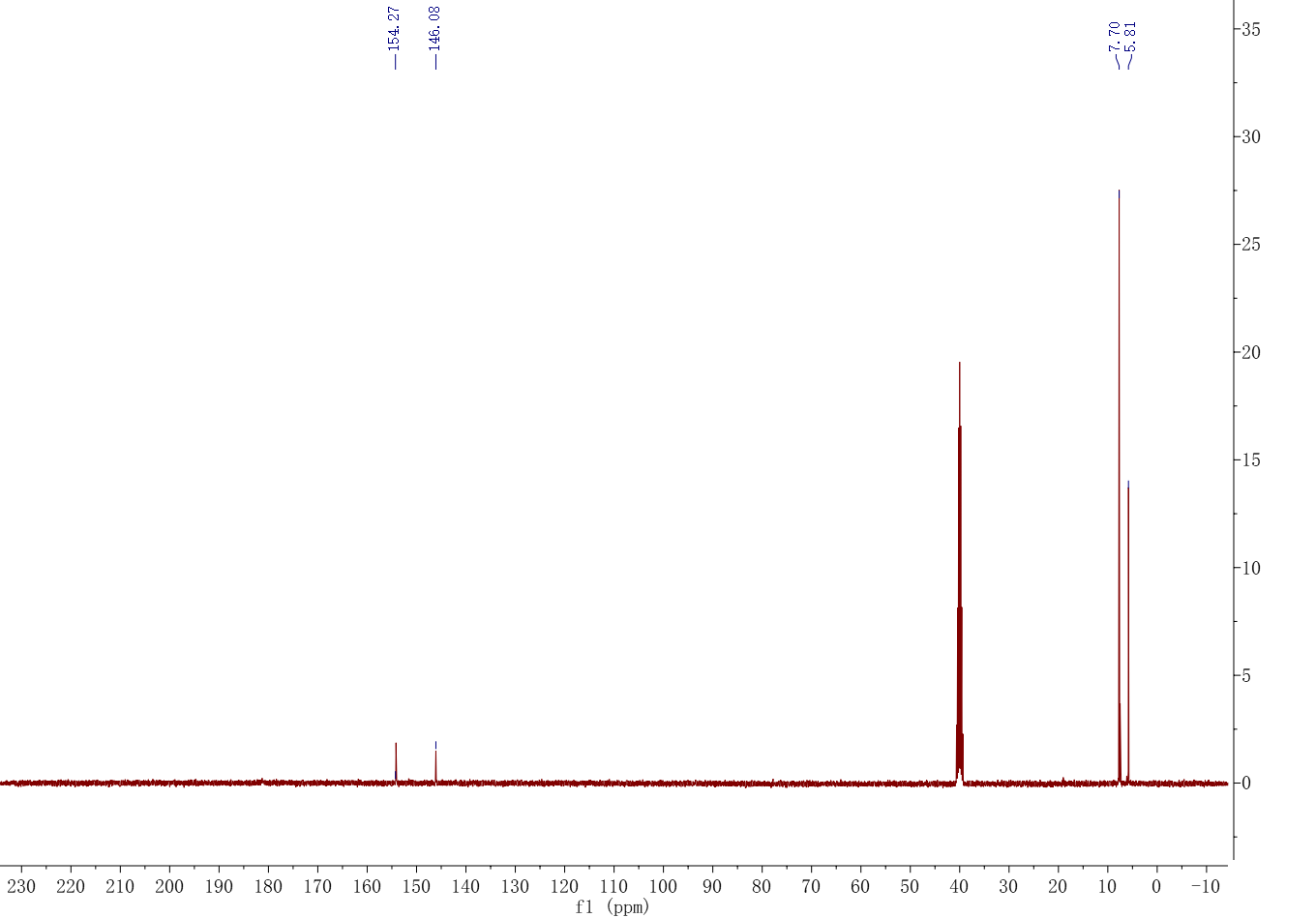


Fig. *^13^C NMR of* **C2** (100 MHz, DMSO)


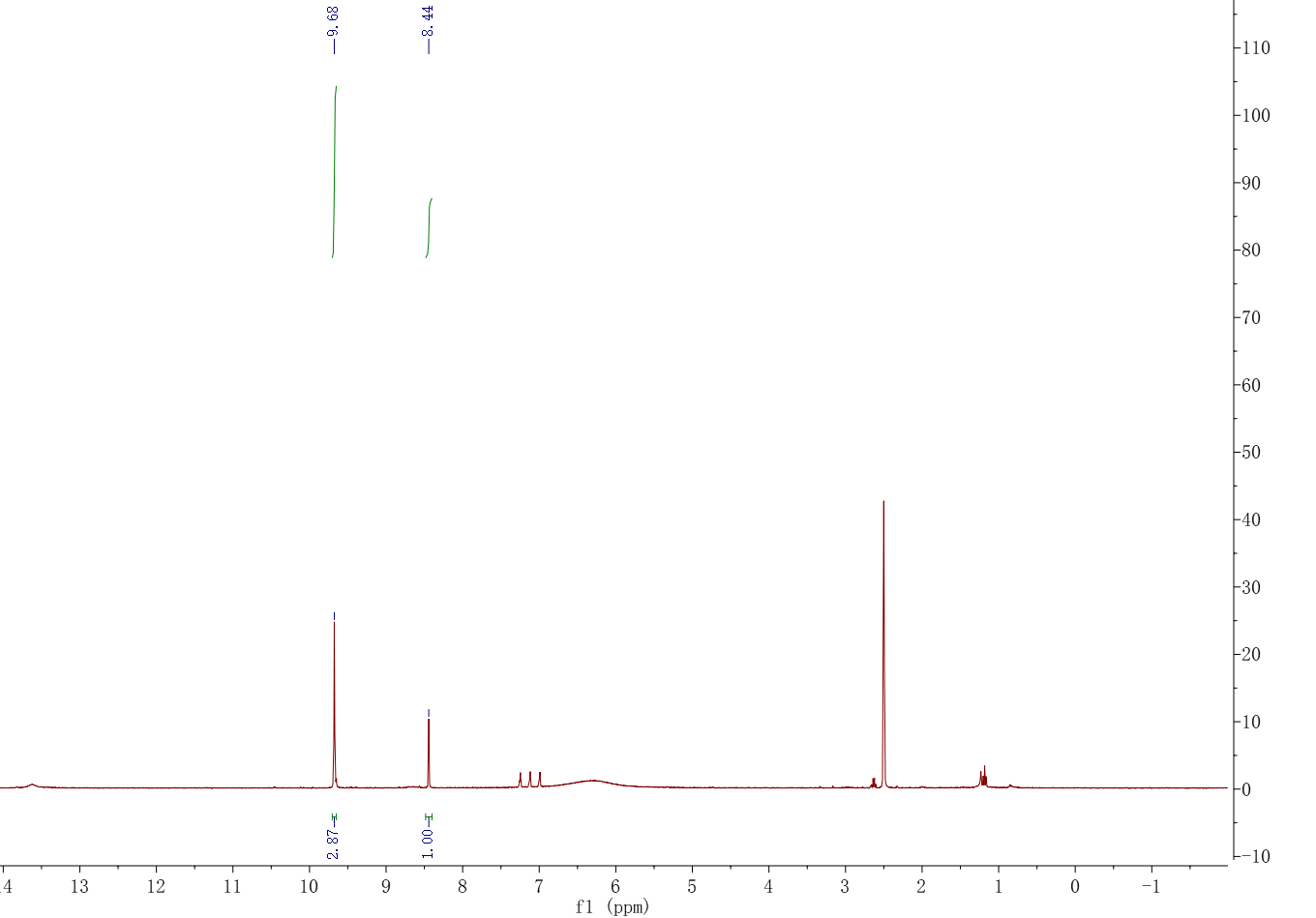


Fig. *^1^H NMR of* **C3** (400 MHz, DMSO)


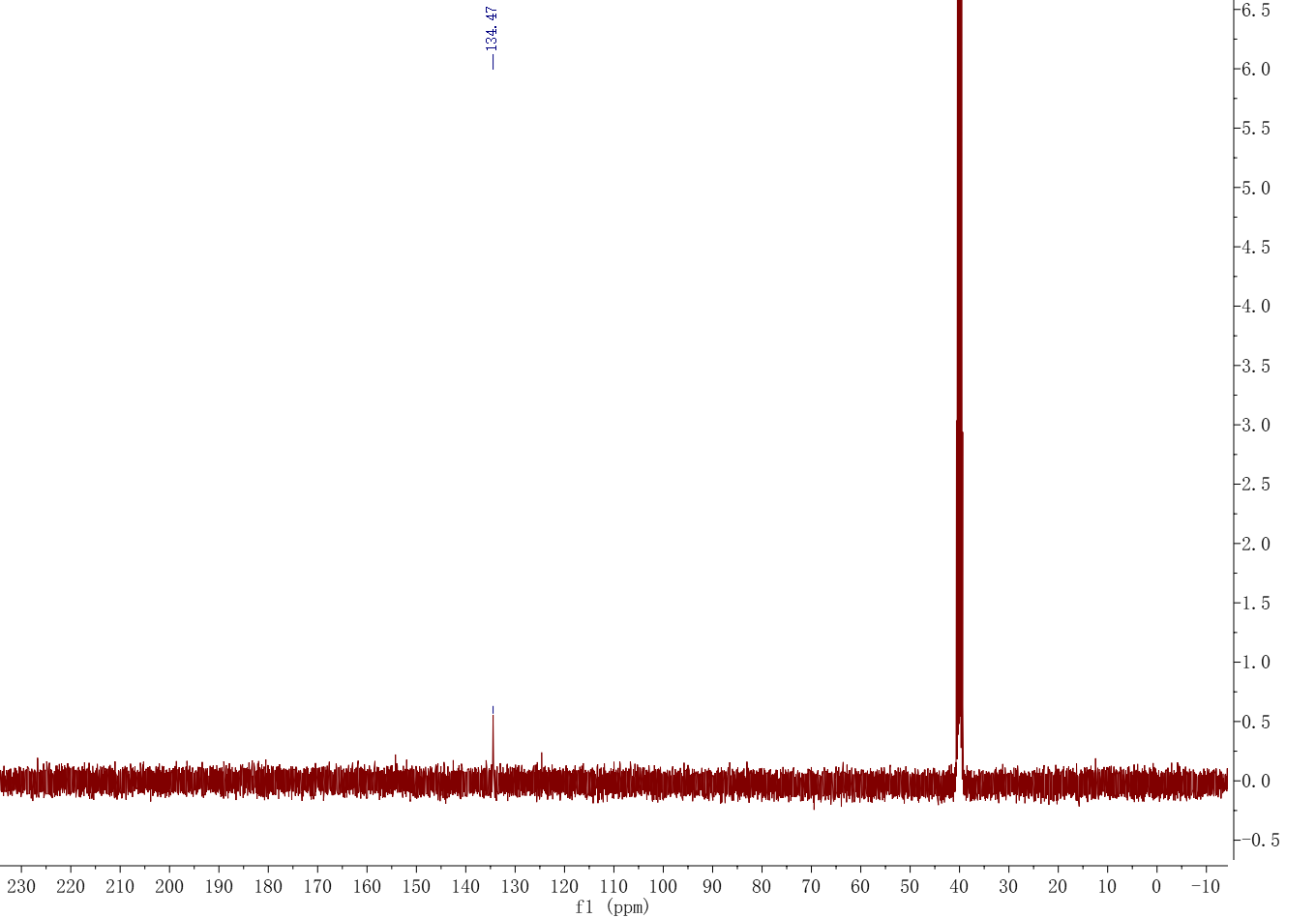


Fig. *^13^C NMR of* **C3** (100 MHz, DMSO)


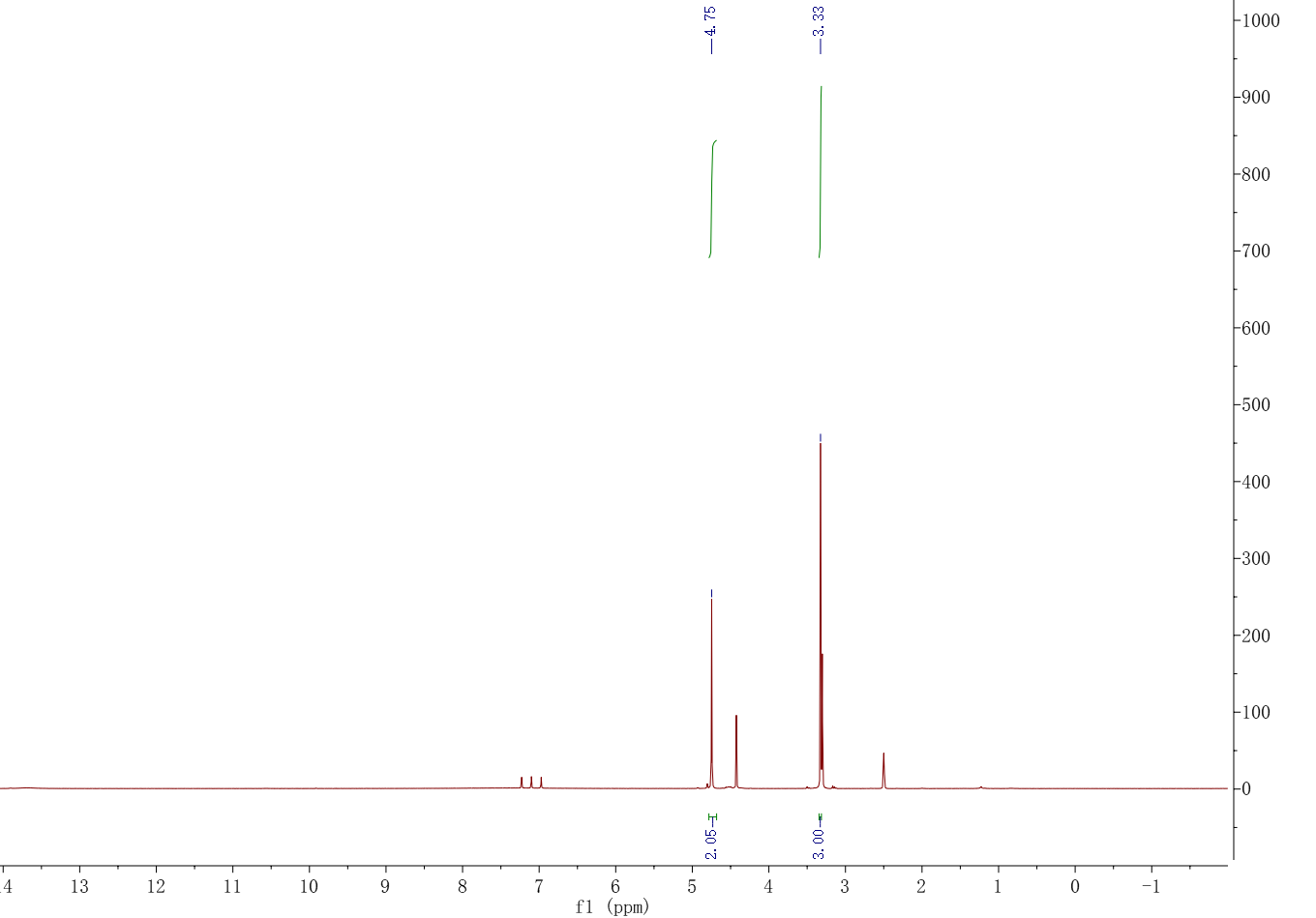


Fig. *^1^H NMR of* **C4** (400 MHz, DMSO)


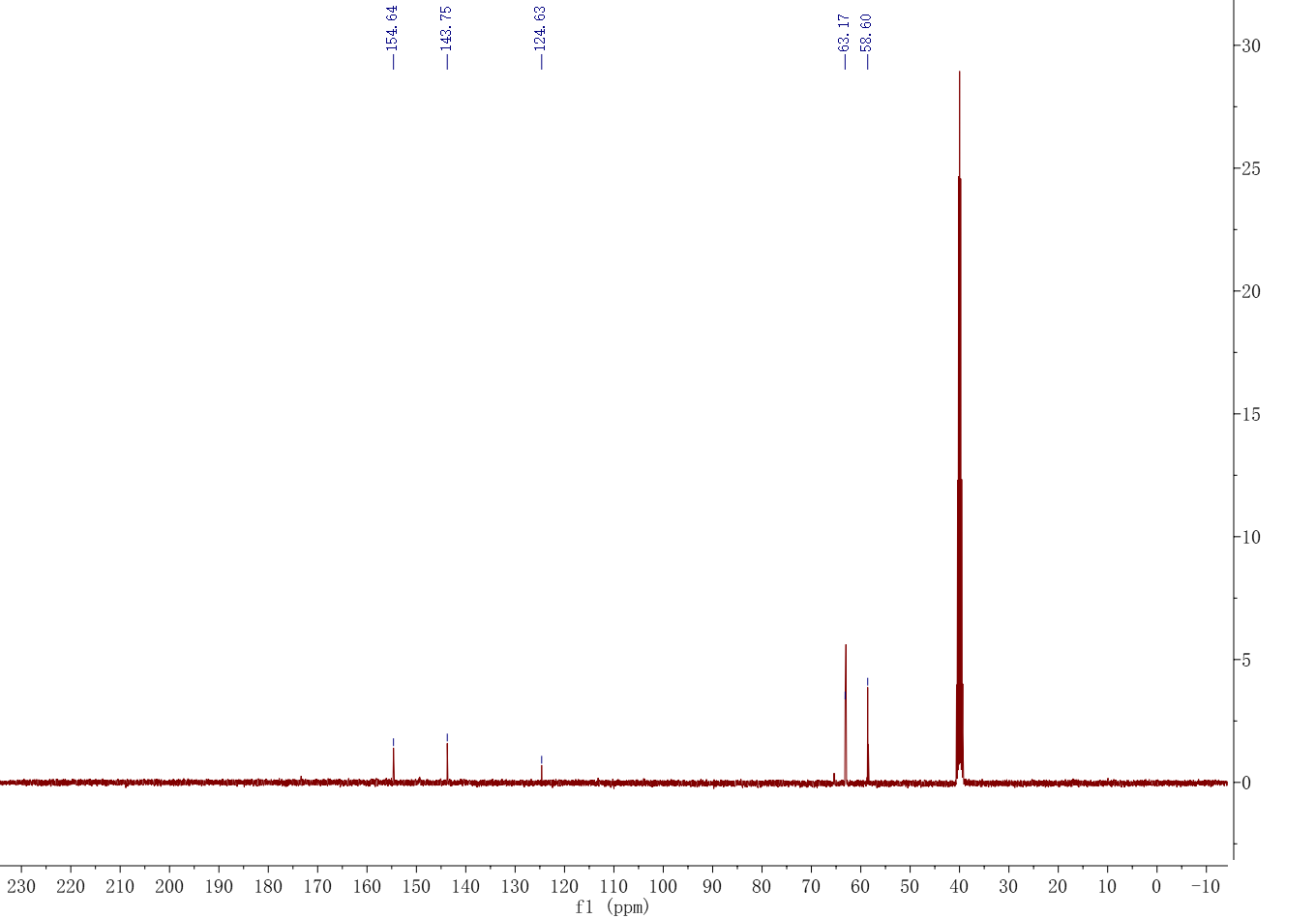


Fig. *^13^C NMR of* **C4** (100 MHz, DMSO)


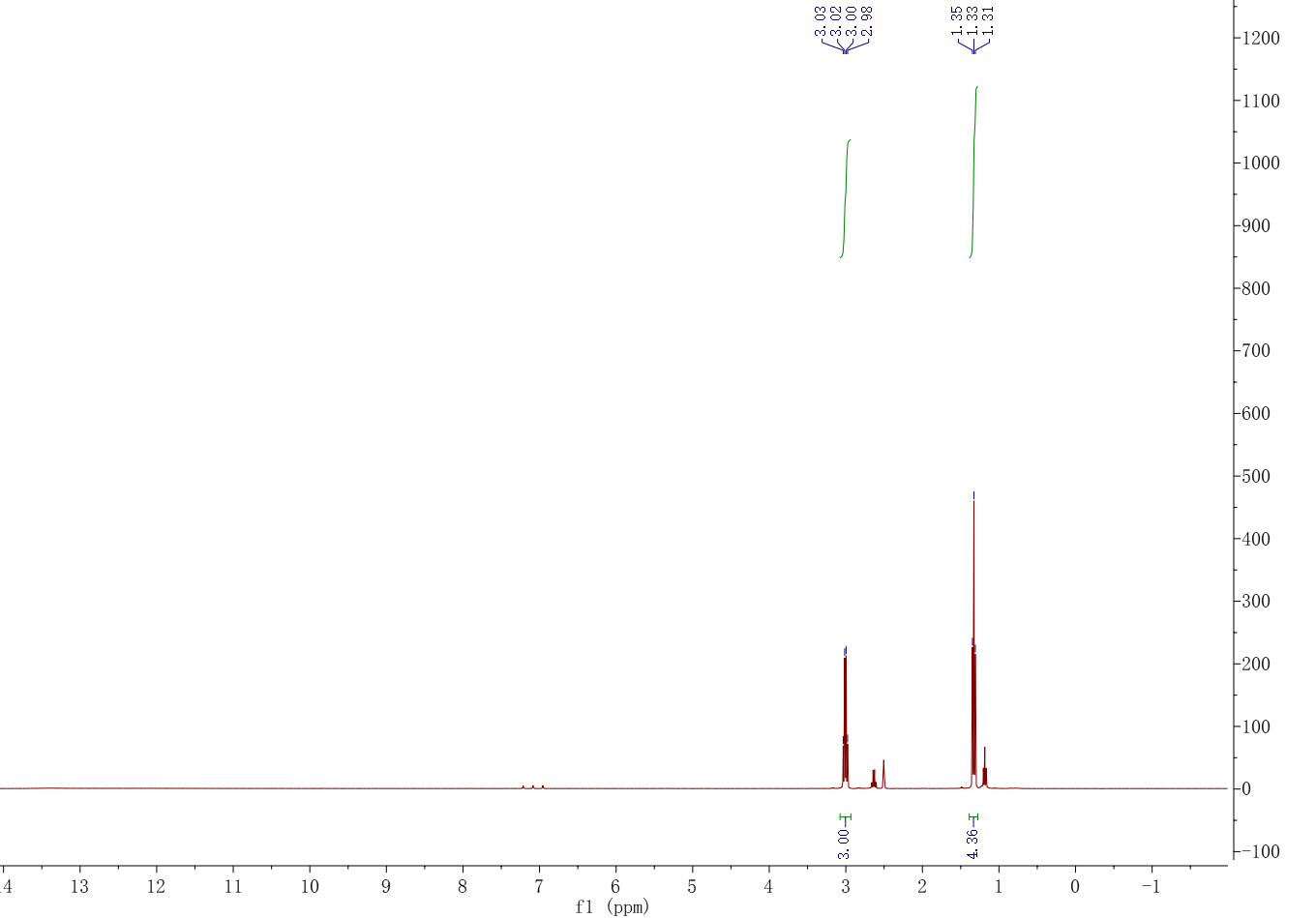


Fig. *^1^H NMR of* **C5** (400 MHz, DMSO)


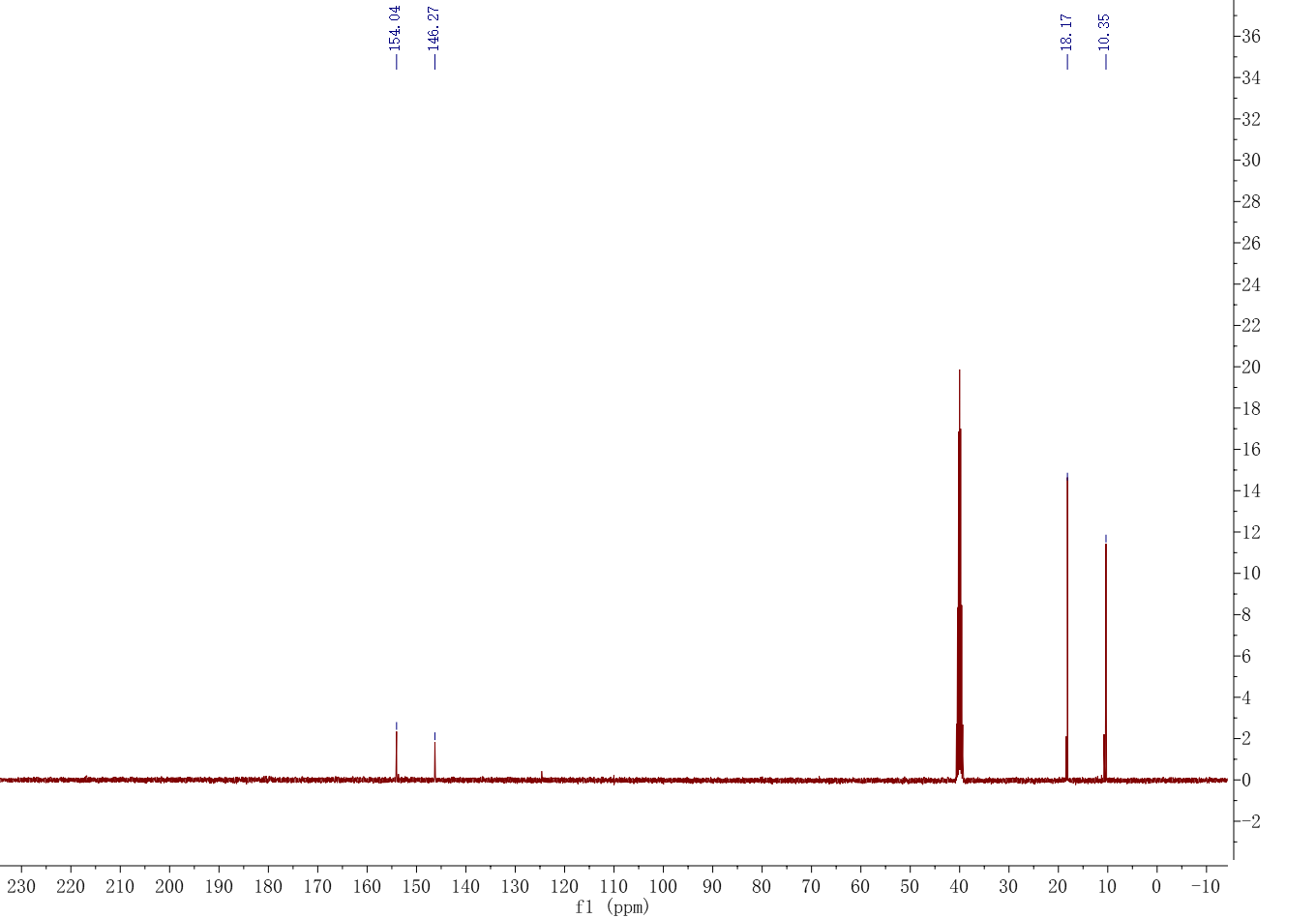


Fig. *^13^C NMR of* **C5** (100 MHz, DMSO)

1. *Correspondent. E-mail: 164013089@qq.com [↑](#footnote-ref-2)
